# Weaning differentially affects the maturation of piglet peripheral blood and jejunal Peyer’s patches

**DOI:** 10.1038/s41598-022-05707-9

**Published:** 2022-01-31

**Authors:** Federico Correa, Diana Luise, Paolo Bosi, Paolo Trevisi

**Affiliations:** Department of Agricultural and Food Sciences (DISTAL), University of Bologna, Viale G. Fanin 46, 40127 Bologna, Italy

**Keywords:** Developmental biology, Immunology, Microbiology, Molecular biology, Structural biology, Systems biology, Gastroenterology

## Abstract

The study aimed to assess how the post-weaning condition changes piglet peripheral blood (PB) and jejunal Peyer’s patches (JPPs) as compared to the suckling period, and how these changes are associated with intestinal microbiota evolution. Sixteen pigs were slaughtered and sampled for PB, JPPs and jejunal content (JC) at weaning (26 days) or at 12 days fed on a pre-starter diet. The PB and JPP transcriptomes were analysed using mRNA-seq. The Gene Set Enrichment Analysis was used to demonstrate enriched gene clusters, depending on sampling time. Jejunal microbiota was profiled using 16S rRNA gene sequencing. Post-weaning JPPs were enriched for processes related to the activation of IFN-γ and major histocompatibility complex (MHC) class I antigen processing which clustered with the reduced abundance of the *Weisella* genus and *Faecalibacterium prausnitzii* in JC. The post-weaning microbiome differed from that seen in just-weaned pigs. For just-weaned PB, the enrichment of genes related to hemoglobin and the iron metabolism indicated the greater presence of reticulocytes and immature erythrocytes. The JPP genes involved in the I MHC and IFN-γ activations were markers of the post-weaning phase. Several genes attributable to reticulocyte and erythrocyte maturation could be interesting for testing the iron nutrition of piglets.

## Introduction

The weaning period is a turning point in the life of commercial swine; the latter must be prepared for the robustness achieved during the suckling phase in order to avoid an excessive decrease in feed intake, transient gut inflammation and intestinal dysbiosis. Weaning implies providing a solid diet based on feeds of vegetal origin and the mixing of pigs from different litters. Thus, the gut of the newly weaned pig faces large quantities of poorly known molecules of dietary origin and of microorganisms scarcely encountered in its previous life. Suckling pigs progressively educate their mucosal lymphoid tissue^1^ upon the multiplication of microorganisms favoured by the presence of milk and, later, of additional offered feed. This is achieved in interdependence with the maturating activity of bone marrow and the thymus, and seeding from the peripheral blood (PB)^2^.

The local porcine immune system is based on: (1) both a diffuse system, mainly in the lamina propria, and on intraepithelial lymphocytes, (2) organised compartments: Peyer’s patches (PPs) which are bud-shaped in the jejunum and continuous single structures in the ileum, and mesenteric lymph nodes; PPs have an important role in the host-microbiota cross-talk. Based on studies regarding surgical removal in rats^3^ and gene knockout mice^4^, PPs are necessary for the production of immunoglobulin A (IgA) and to avoid intestinal pathogen translocation^5^. In the lumen-protruding epithelium associated with PP follicles, specialised cells collaborate to detect and deliver particles from the lumen to the underlying tissue. B cells proliferate in the germinal centre of PPs, have somatic hypermutation and undergo selection upon the induction of T cells which are primed in the PP interfollicular area. Some recent evidence has finally suggested that ileal PPs (IPPs) initiate their development prenatally and accelerate after birth^6^, being more involved in primary, undiversified IgA production^7^ while jejunal PPs (JPPs) develop postnatally, being more responsible for initiating the production of diversified IgA.

Recent reviews have addressed the evolution of the gut microbial profile in piglet early life^8,9^. However, the major part of the microbiota data were not obtained from samples of the small intestine and did not compare the variability of the microbiota in the suckling pig with the maturation of PPs located in the second half of the small intestine. The need still exists to assess how the variations of the microbiota in the small intestine after the weaning period compare with those induced in PPs and with pre-weaning values. The availability of a modern sequencing method to detail the transcriptome and microbiota gives important opportunities to disclose the involvement of under-considered biological pathways and potential marker genes. The PB is also an interesting area of study due to the minor invasiveness of the sampling and the possibility of replicating on the same individual. Their availability could speed the assessment of the efficiency of the dietary or management strategies proposed for the piglet in the transition from late suckling to the weaning period, and thereafter.

The general aim was to increase knowledge regarding the connection between the colonisation of the small intestine, the development of local immunity in piglets and their general health. The specific aim was to focus on the transcriptome profiles of JPPs and PB in healthy pigs at the time following separation from their mothers and at 12 days post-weaning, and to connect these results with the simultaneous variation of the microbiota in the jejunum.

## Results

**Figure 1:**
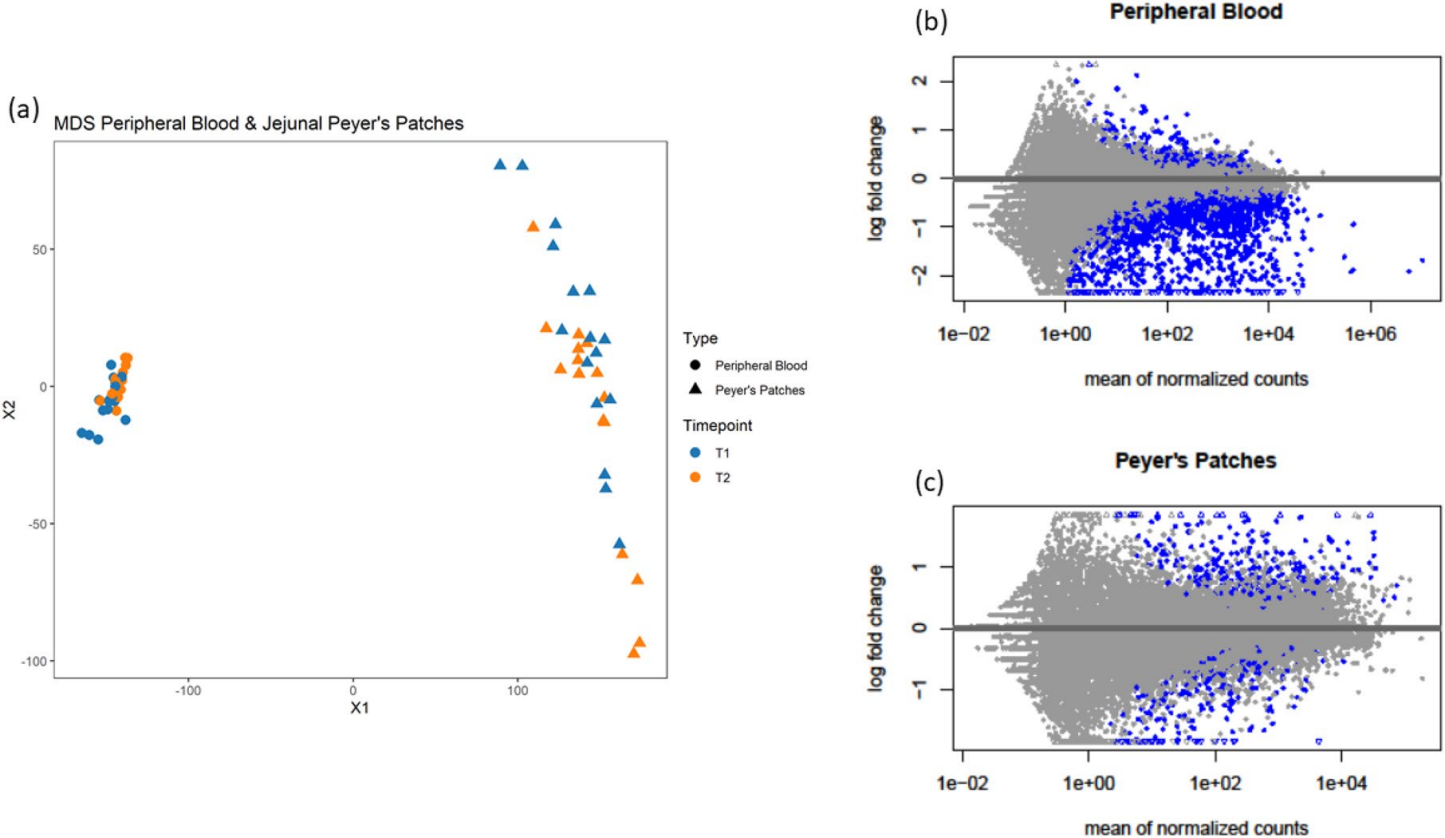
Summary plots of the gene expression profile of JPPs and PB at different times. The plots were multidimensional scaling (MDS) plots for the normalised gene expression levels of JPPs and PB at weaning (T1) and at 12 days post-weaning (T2) (**a**), and smear plots of the differential expression of the genes between T1 and T2 for JPPs (**b**) and PB (**c**), respectively. The smear plots show the relationship between the log fold change and the mean normalised count. The grey points represent non-significant differentially expressed genes whereas the blue points show genes which are significantly differentially expressed.

For PB and JPPs, 21,424 and 22,953 transcribed genes were recovered, with a mapping rate of 79 ± 3% and 78 ± 3%, respectively. The genes with useful attribution were 15,509 and 16,197, respectively. The separation between tissues was evident using preliminary multidimensional scaling plots. The JPP samples did not seem to cluster based on the timepoint; instead, the PB samples tended to cluster better (Fig. 1a). Smear plots (Fig. 1b,c) showed that the majority of genes were centred around a log fold change (FC) of zero indicating that any composition bias between libraries was successfully removed.

### Effect of weaning on differential gene expression analysis in JPPs

**Figure 2:**
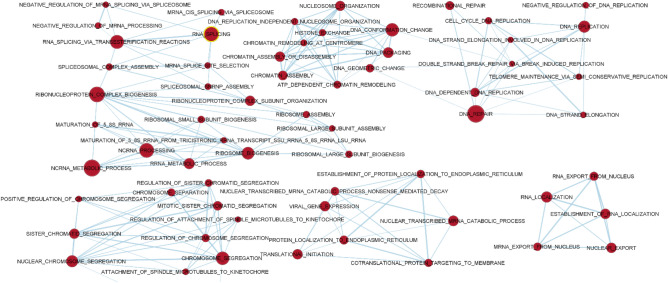
Gene sets upregulated in jejunal Peyer’s patches of pigs on the day of weaning as compared to those sampled in pigs at 12 days post-weaning. The sets are enriched with FDR < 0.01. The edges represent the link of two or more gene sets sharing the same core group of genes, explaining the enrichment of each of the gene sets. Node colour intensity conveys enrichment significance (P-value) while their dimension increases with the number of their genes.

The JPPs sampled at weaning and at 12 days post-weaning presented 114 and 31 significantly enriched gene sets (False discovery rate [FDR] < 0.05), respectively. The transcriptome of the JPPs of the just-weaned pigs demonstrated intensive proliferative activity as compared to that at 12 days post-weaning. In fact, several gene sets related to DNA replication, elongation and repair; chromosome segregation; chromatin and histone changes; mRNA export, regulation, maturation and ribosome activity, and translation activation were more enriched in JPPs at weaning (Fig. 2).

**Figure 3:**
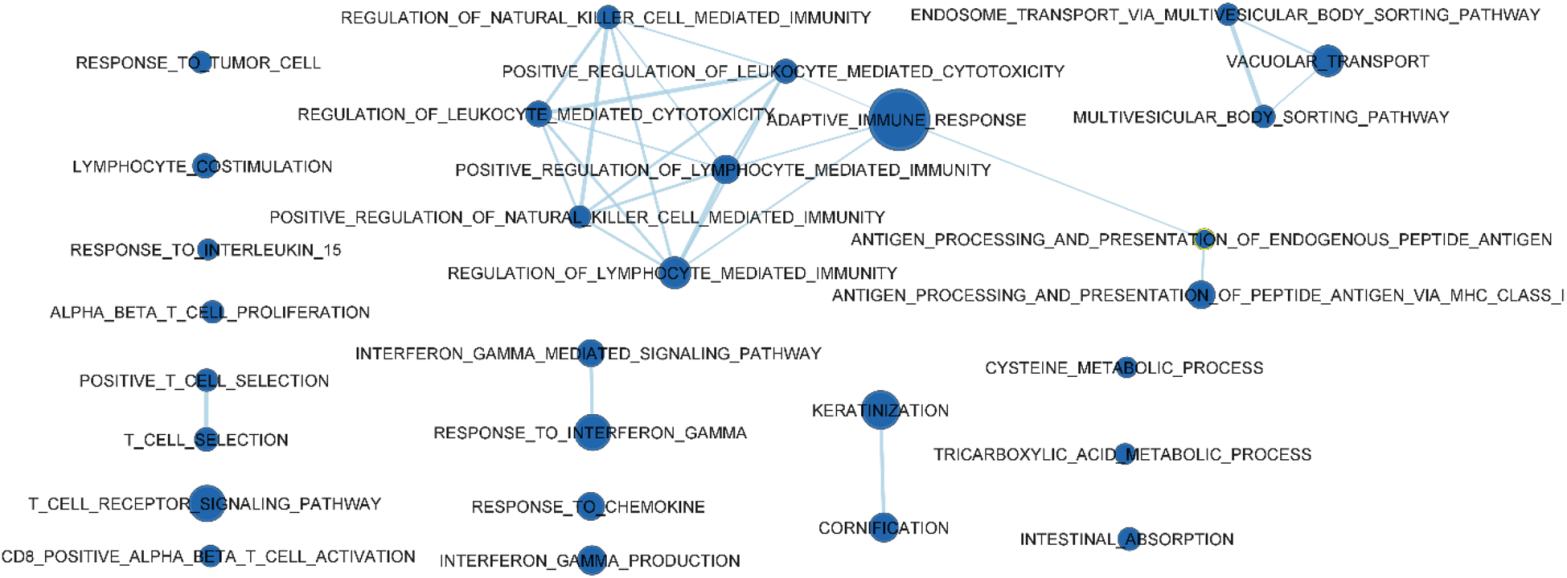
Gene sets upregulated in jejunal Peyer’s patches of pigs sampled at 12 days post-weaningas compared to those on the day of weaning. The sets are enriched with FDR < 0.05. The edges represent the link of two or more gene sets sharing the same core group of genes, explaining the enrichment of each of the gene sets. Node colour intensity conveys enrichment significance (P-value) while their dimension increases with the number of their genes.

Conversely, in post-weaning JPPs, the gene sets were mainly related to immune organisation and local structure, and the functions were enriched (Fig. 3).

Biological processes related to interferon gamma (IFN-γ) production and response were involved, with a particular impact on the activation of the processing of major histocompatibility complex (MHC) class I antigens and their presentation. This, in turn, influenced several types of immune cells, including CD8+ cells and natural killer cells. Other enriched gene groups potentially related to immune functions were those related to the response to interleukin (IL) 15 and multi-vesicular body transport in cells. The transcriptome for structural organisation of the JPPs was more differentially affected by the enrichment of genes associated with keratinisation and cornification of cells. Genes for the metabolic process of cysteine and tricarboxylic acids were also upregulated.

The JPPs sampled at weaning and at 12 days post-weaning presented 126 and 164 genes positively differentially expressed, respectively (Supplementary Table 1). Melanotransferrin was the most upregulated gene at weaning and subtilisin-like proprotein convertase (*PCSK9*) at 12 days post-weaning.

### Effect of weaning on differential gene expression analysis in the PB

**Figure 4:**
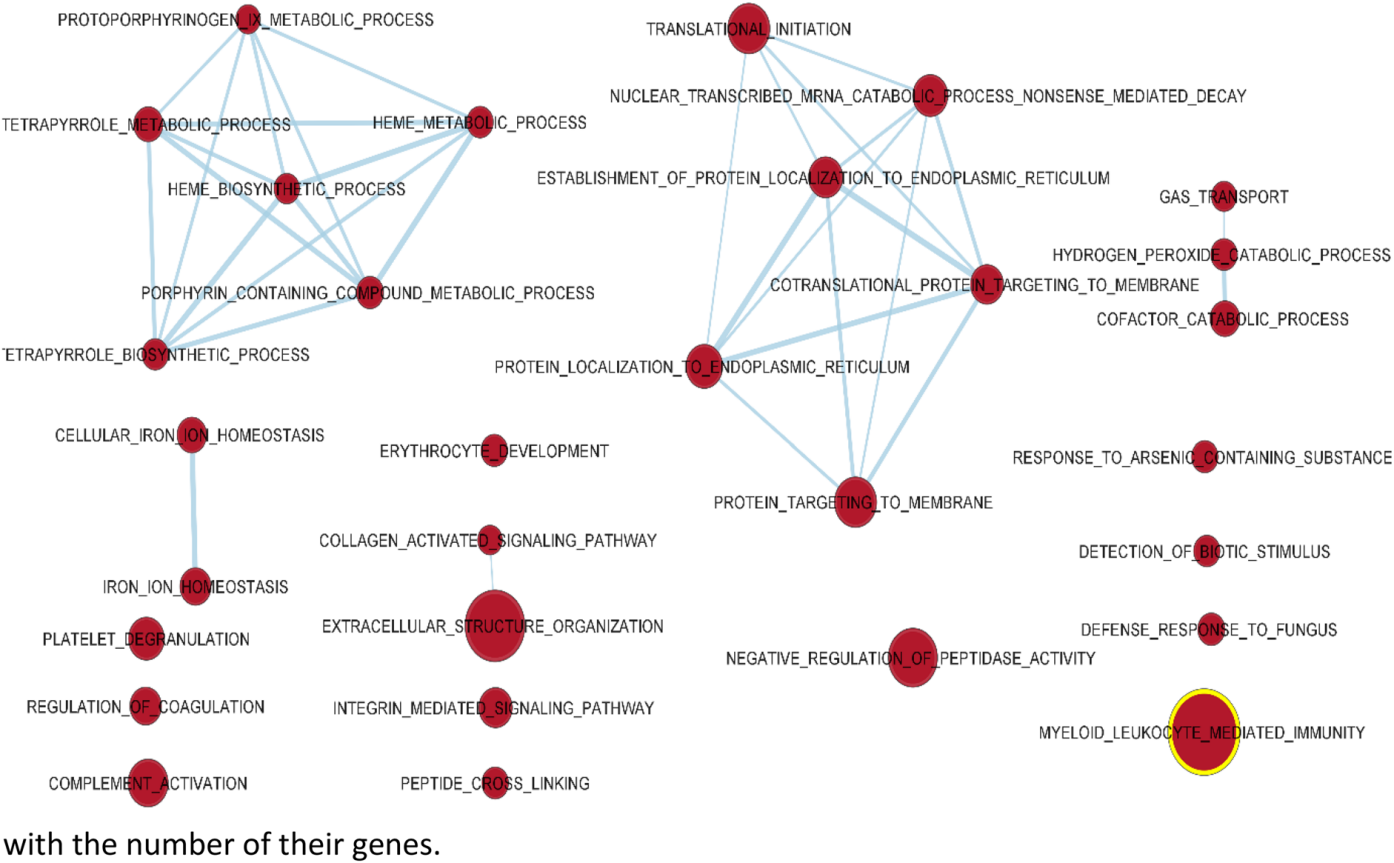
Gene sets upregulated in the peripheral blood of pigs on the day of weaning as compared to those sampled in pigs at 12 days post-weaning. The sets are enriched with FDR < 0.01. The edges represent the link of two or more gene sets sharing the same core group of genes, explaining the enrichment of each of the gene sets. Node colour intensity conveys enrichment significance (P-value) while their dimension increases with the number of their genes.

The PB sampled at weaning and at 12 days post-weaning presented 114 and 31 enriched gene sets (FDR < 0.05). In PB of pigs at weaning, Gene Set Enrichment Analysis (GSEA) evidenced primarily the enrichment of gene sets related to haeme biosynthesis and the metabolic process as compared to post-weaning (Fig. 4). This was also associated with other sets typical of the PB: erythrocyte development, iron haemostasis, and regulation of coagulation. Other enriched sets were those related to the protein translation and post-translational processes.

**Figure 5:**
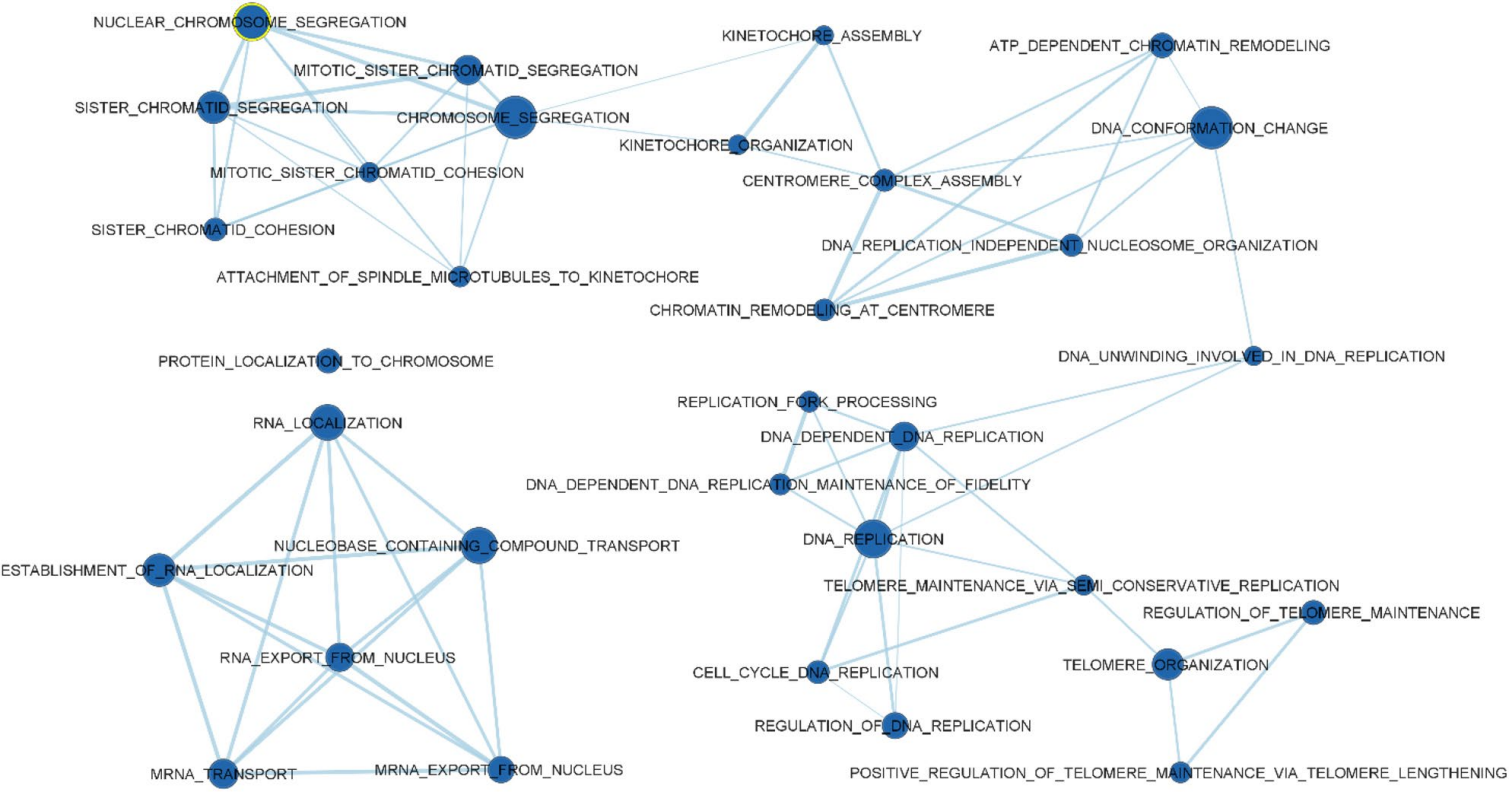
Gene sets upregulated in the peripheral blood of pigs sampled at 12 days post-weaning as compared to those on the day of weaning. The sets are enriched with FDR < 0.01. The edges represent the link of two or more gene sets sharing the same core group of genes, explaining the enrichment of each of the gene sets. Node colour intensity conveys enrichment significance (P-value) while their dimension increases with the number of their genes.

The PB at weaning and at 12 days post-weaning presented 1195 and 242 up-regulated genes (Supplementary Table 2). Of those more expressed at weaning, there was haemoglobin subunit beta-like (ENSSSCG00000014727), not included in the list of gene sets. Bisphosphoglycerate mutase was the most upregulated gene at weaning (Padj = 2.9 × e^−9^). Peptide YY (*PYY*) and arginase 1 were among the genes not directly associated with haemoglobin function which were most upregulated at weaning (Fig. 5).

### Effect of weaning on the microbiota of the jejunal content

**Figure 6:**
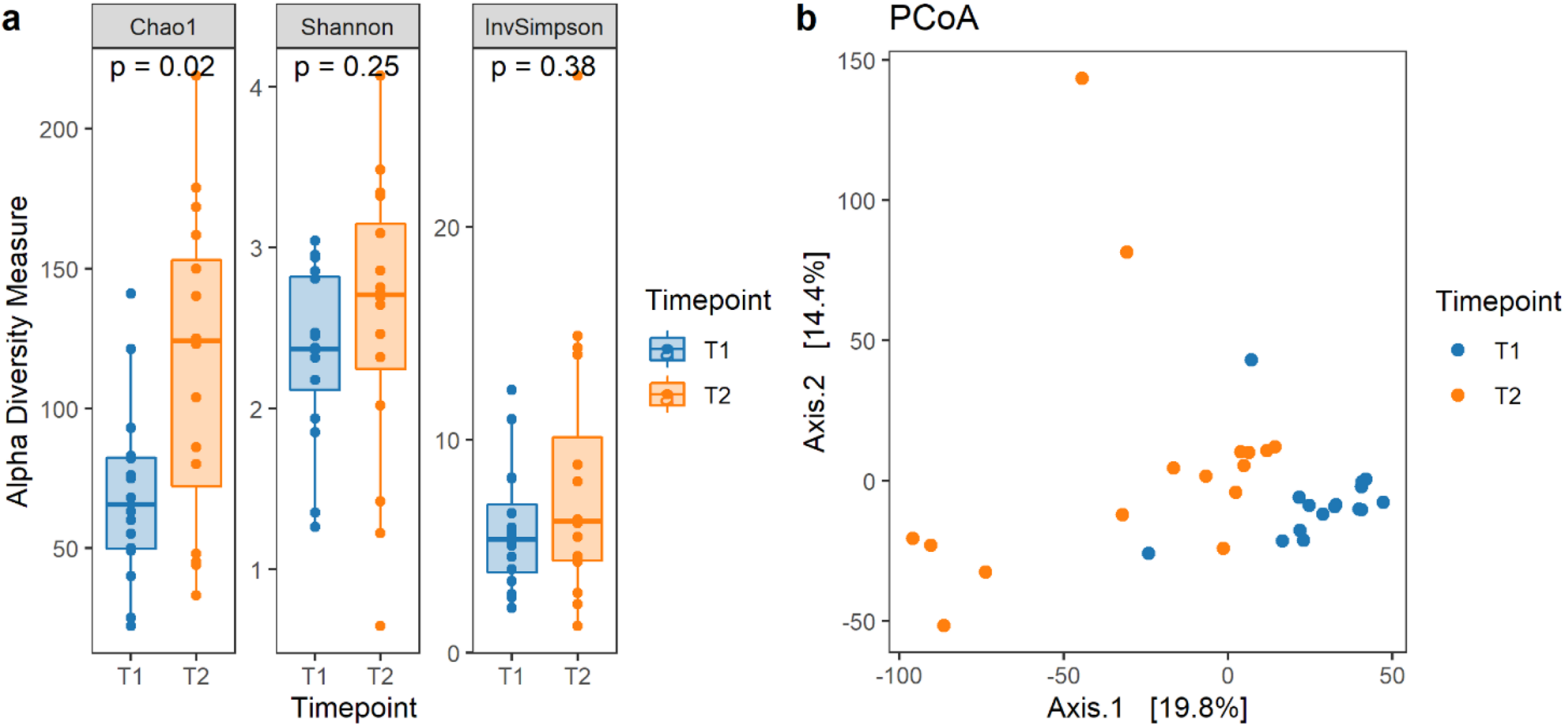
Descriptive plots for bacterial diversity. (**a**) Boxplots of the Chao1, Shannon and InvSImpson α-diversity for each timepoint. (**b**) Non-metric multi-dimensional scaling (NMDS) plot using a Euclidian distance matrix for the 32 samples coloured according to timepoint.

The sequencing procedure of the jejunal content (JC) produced 46,109 sequences on average per sample and a total of 882 amplicon sequence variants (ASVs). The ASVs were aggregated into 21 phyla, 96 families and 179 genera. The most abundant phylum was Firmicutes. accounting for 92.8 ± 5.3% of the total (mean ± d.s.) at both time periods, followed by Actinobacteriota 6.6 ± 1.4%. Lactobacillaceae and Streptococcaceae were the two most abundant families, accounting for 70.8 ± 8.2% and 14.3 ± 4.6%, respectively. Lactobacillus, 67.9 ± 7.6%, and Streptococcus, 14.3 ± 4.7%, were the most abundant genera. At 12 days post-weaning, the bacterial richness (Chao1) increased (Fig. 6-a); this effect was not observed for the other measurementss used (Shannon and InvSimpson). For beta diversity, weaning affected the jejunum bacteria composition (R^2^ = 0.13, *P* = 0.001), as is also shown by the principal coordinates analysis (PCoA) plot in Fig. 6-b, in which samples are clustered based on timepoint.

Several genera were enriched in the JC at 12 days post-weaning (Supplementary Table 3). The increased abundance of *Streptococcus porcorum*, *Collinsella aerofaciens* and of an ASV belonging to the *Blautia* genus was relevant for 4.28; 4.33; 8.74 Log2 Fold Changes, respectively as compared to the JC at weaning.

### Effect of the clustering of weaned pigs of MHC class I antigens in JPPs on the jejunal microbiota

Due to the relevance of activation of the response of MHC class I antigens and IFNγ in JPPs sampled in the post-weaning phase, the post-weaning pigs were clustered into two groups differing as to the expression of 44 genes relevant to this response. The normalised expression of genes for cluster group 1 was, in general, higher than that of cluster group 2 (Supplementary Table 4). No effect of the attribution of each pig to the specific cluster group on Alpha and Beta-diversity of the jejunal microbiota was observed (Supplementary Figs. 1 and 2). However, the pigs classified using statistical analysis as belonging to the cluster group with the higher activation of response to MHC class I antigens and IFNγ presented a highly significant reduction in the presence of two AVSs, belonging to the *Weissella* genus (mean abundance, cluster 1 = 0; cluster 2 = 398.99, Log2 Fold Change = − 11.1; adjusted P = 0.00019) and *Faecalibacterium prausnitzii* (mean abundance, cluster 1 = 0; cluster 2 = 42.47; Log2 Fold Change = − 23.13; adjusted P = 3.5 × e^−15^).

## Discussion

### Effect of weaning on the differential activations of MHC class I antigens in JPPs

Early stimulation by diverse microbiota acquired in the environment is important for developing the ability of inducing the complex activation of MHC in pigs to expand the intestinal immune system upon the encounter of different non-self antigens^10^. Compared to that of the just-weaned, the post-weaning condition of piglets was marked by switching to the activation of JPPs, related to the adaptive response associated with MHC class I stimulation and also to cells less dependent on antigen activation, such as natural killer cells^11^. This observation fits with the delayed maturation of CD8+ T cells^12^ which are also located in the PP inter-follicular areas^13^ and stimulated by class I antigens in the porcine jejunal lamina propria up to five weeks of age. Post-weaning did not affect the response related to MHC class II which was associated with the observation that CD4+ positive cells were typically stimulated by the molecules of that class and developed earlier in the small intestine of pigs^12^. Furthermore, comparing the transcriptome of the IPPs of pigs still suckling at 21 days of age with those obtained from pigs weaned at this age but then reared for another week, the pathway Immune response MHC class I antigen presentation was positively affected^14^. Conversely, Immune response MHC class II antigen presentation was negatively affected^14^.

**Figure 7:**
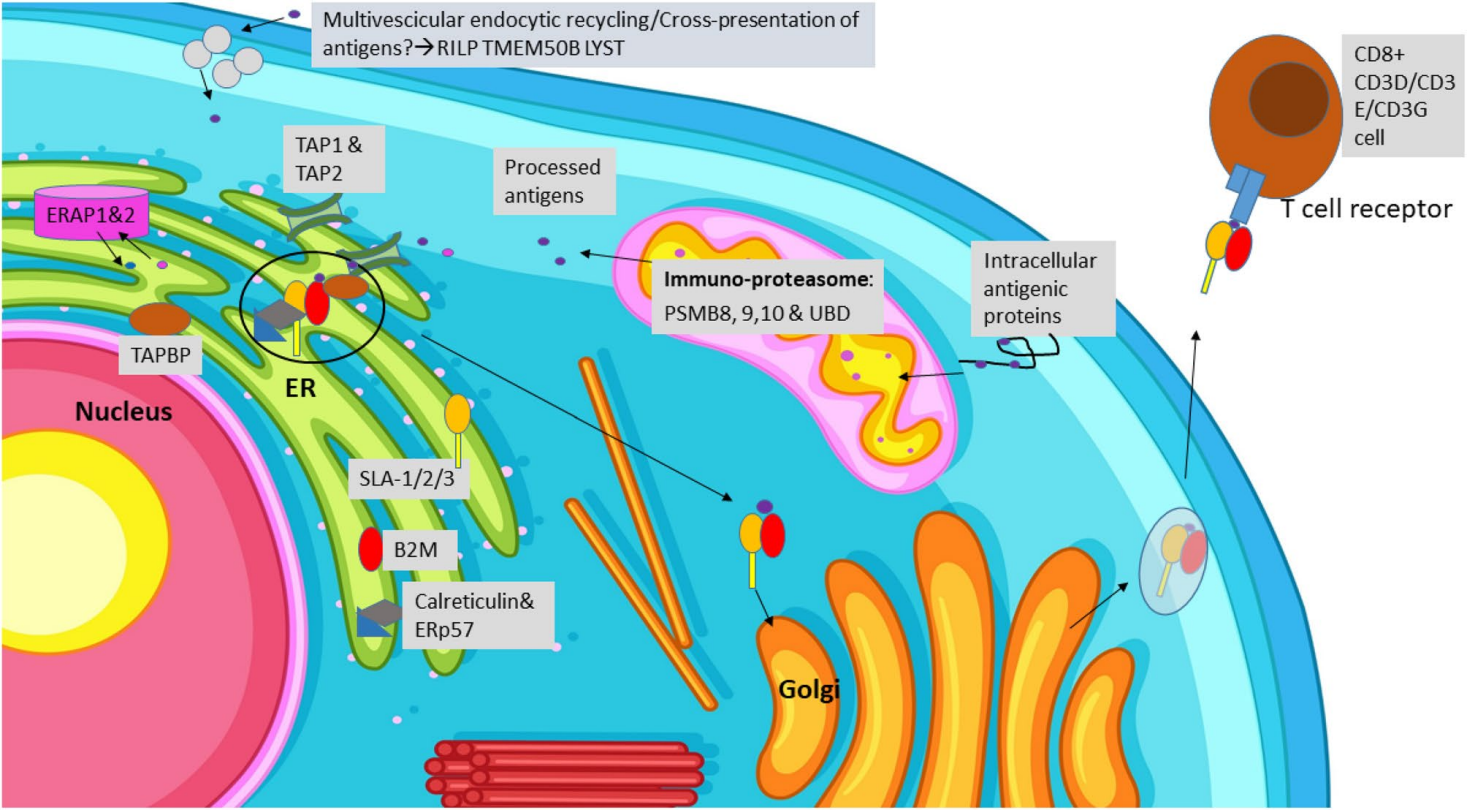
Scheme of the process related to MHC I activation upregulated in post-weaning pigs. Upregulated genes, alone or inside enriched groups, are in the figure in capital letters. In activated cells, such as DCs, a special proteasome complex is formed, thanks to 20S Subunit Beta 8, 9 and 10 (PSMB8/9/10) and ubiquitin D (UBD), to process intracellular antigenic proteins. Cross-presentation of external antigens (from neighbouring cells, cell fragments) can be processed using multivescicular body sorting (*RILP* Rab Interacting Lysosomal Protein, *TMEM50B* Transmembrane Protein 50B, *LYST* lysosomal Trafficking Regulator). Entering into the endoplasmic reticulum (ER) by the channel made by Transporter 1 and Transporter 2, ATP Binding Cassette Subfamily B Members (TAP1 and TAP2), accepted antigens are integrated to MHC I (SLA-1, SLA-2, SLA-3) inside the complex, also formed by the TAP Binding Protein (TAPBP), β-2-microglobulin (B2M) calreticulin and ERp57. Some peptides must be preliminarily trimmed by endoplasmic reticulum aminopeptidase 1 and 2 (ERAP1 and ERAP2). The antigen-charged complex B2M—MHC I is then exported to the Golgi apparatus where it is packed in vesicle form to be exported from the cell. Design created in Microsoft PowerPoint 2016, and a part of the personal design freely obtained from http://www.freepik.com.

A complex array of phagocyte cells from different lineages of myeloid origin, under the general name of dendritic cells (DCs)^15^ were aligned in the PP area. Some of them were located more in the interfollicular area, in the proximity of the aggregation of CD8+ T cells. These DCs play an important role of presenting other types of antigens, mainly of endocellular origin, priming naïve CD8+ T cells to polarise into cytotoxic T lymphocytes, in a local environment which is enriched by the secretion of IFN-γ from already primed T cells^16^. The molecular mechanism for processing antigens into the MHC I system is a kit which is the heritage of all nucleated cells; however, in PPs, a specific context is found for this process. The diffuse enrichment of several groups of genes involved in MHC class I activation after weaning in JPPs merits more specific discussion (Fig. 7).

Weaning up-regulated almost all the genes associated with antigen processing and the presentation of the endogenous peptide antigen (Fig. 7), including those coding for three subunits of porcine MHC class I (Swine Leukocyte Antigens, *SLA-1*, *SLA-2*, *SLA-3*)^17^. Other genes were those promoting entrance into the ER (*TAP1* and *TAP2*), the invariant light chain stabilising MHC class I, β-2-microglobulin (*B2M*), and the *TAPBP*; the eventual preliminary trimming of some peptides is carried out by the ER aminopeptidases (*ERAP1* and *ERAP2*).

In any cell, the majority of the antigenic molecules to be processed require reduction inside the proteasome complex. However, upon promotion by IFN-γ, this complex is integrated by three catalytic subunits (β1i, β2i and β5i) to form a immuno-proteasome^18^, specialised in the trimming of antigens for MHC I presentation. In post-weaning, the genes regulating the IFN-γ response were upregulated (IFN-γ is diffuse particularly in the interfollicular PP environment), and the three genes coding for the immuno-proteasome subunits (*PSMB8*, *PSMB9* and *PSMB10*) were also upregulated (Fig. 7). Another gene (*UBD*), coding for a protein-like ubiquitin, the main actor starting the proteasome process, was also over-expressed. These observations suggested that the immuno-proteasome in JPPs maturates more after weaning, to improve the antiviral adaptive immune responses against intracellular infections. It could be proposed that *PSMB8*, *PSMB9* and *PSMB10* are important markers for testing JPP maturation.

The enrichment of the gene set related to expansion by the cell division of the T cell populations bearing the αβ type receptor, together with the upregulation of CD8A and several genes forming the T-cell antigen receptor complex (CD3D/CD3E/CD3G), additionally supports the involvement of CD8+ T cells (Fig. 7) as a target of MHC I activation in post-weaning pigs. Interestingly, interleukin 15 (*IL-15*) and its receptor (*IL15RA*) were also upregulated in the post-weaning period. The maturation and survival of CD8 cells is promoted by IL-15, by the link to IL-15 receptors on these T cells^19^.

Antigens accessible to MHC class I can derive from proteins of the viruses or microbes infecting the cells or were phagocytised. However, DCs can form a junction with infected non-immune cells and obtain fragments to process from them^20^, producing a cross-presentation and priming. It is possible that this process was more stimulated after weaning because the gene set related to multivesicular endocytic recycling was one of the most enriched (First genes: *RILP, TMEM50B, LYST*). In the context of JPPs it is also possible that mucosal antigens are transported by IgA to activate local DCs and prime T cells^21^. Finally, the need to recycle relevant quantities of MHC I molecules could explain the 1st ranking of *PCSK9*, involved in their complexation and additional lysosomal degradation^22^.

The position of JPPs is strategic for sampling and controlling the proximal lumen. Emerging microbes could be useful in supplementing pigs in order to specifically orient the immune response in this phase. There was scarce evidence that membership of the pigs in the group with more activation of these pathways differed in the microbiota profile. However, the presence of the *Weissella* genus and *Faecalibacterium prausnitzii* apparently prevented the maximal activation of these pathways. *Faecalibacterium prausnitzii* is a member of Clostridium cluster IV, abundant in the human intestine in which it contributes to maintaining gut health by fermenting fibre and producing volatile fatty acids^23^. It has been isolated in pigs since 2014^24^ and its presence in the gut content characterised the effect of supplementation with bovine colostrum to the standard weaning diet^25^. This microbe is more documented in the porcine hindgut than in the small intestine^26^; thus, in the future, variations of the abundance of this microbe in the jejunums of piglets deserves more attention in order to understand the local activation of the immune system. The *Weissella* genus includes lactic acid bacteria, currently found in the gut of mammals, which are the object of attention as potential beneficial microorganisms due to their ability to produce bacteriocins and also opportunistic pathogens depending on the strain^27^.

### Effect of piglet age and weaning on other gene sets in JPPs

The germinal centres of PPs are important for B cell proliferation and somatic hypermutation, and are inductive sites for IgA^28^. The T-follicular helper cells interact with IgM + naive B cells, inducing the class switching into IgA + B cells^29^. Nevertheless, no effect of age was seen regarding the gene sets related to controlling this process, indicating that, notwithstanding the increase in the volume of the JPPs with age^13,30^. this was not the main inductive site. It is possible that post-weaning affects the B cell receptor-signalling pathway in IPPs, considering that this pathway was up-regulated there as compared to JPPs^31^.

Peyer’s patches are more evident at anatomic inspection as protruding buttons as the pigs age. This requires more structure as evidenced by the presence of enriched gene sets related to cornification and keratinisation. The domes of PPs are marked by the presence of M cells, local cells specialised in the active transepithelial vesicular transport of microorganisms and macromolecules from the lumen to the subepithelial lymphoid tissue. Cytokeratin (KRT) 18 has been proposed as an M-cell marker in porcine PPs^32^. In the present trial, inside these gene sets, the most significant KRTs were *KRT19* and *KRT20*, instead of *KRT18.* This could mean that other KRTs, rather than *KRT18,* are more relevant for the overall structural maturation of PP*s*.

The younger and not yet weaned pigs showed a marked unidirectional pressure towards cell replication, evidenced by a large array of enriched gene sets as compared to older and weaned pigs. Inoue et al.^14^ evidenced that the transcriptome of the IPPs of suckling piglets between 21 and 35 days of age changed very little. Conversely, simultaneously weaned pigs differed sharply from up- and down-regulated genes as compared to unweaned pigs^14^ while the IPP volume increased until 28 days of age in suckling pigs but it was lower in simultaneously weaned pigs^14^. This indicated that, if the suckling period continues, the environmental stimuli tend to be constant and PPs just increase in volume. Thus, it can be hypothesised that this was also the condition of the JPPs in the present study while, in weaned pigs, the antigenic pressure tends to drive the JPPs towards qualitative maturation, as discussed in the previous section.

### Effect of weaning on differential gene expression analysis in the PB

The PB is a very complex tissue due to the presence of cells of different series (erythrocytes, leukocytes, and platelets). These cells have a different origin and time of maturation, and it is not surprising that the changes seen in the PB transcriptome did not resemble those seen in JPPs, notwithstanding the fact that both contain some cells of common origin (immune cells).

Numerically, the dominant cells are erythrocytes which, in pigs, have a life of approximately 72 days^33^; thus, it was not surprising that the genes most represented were those related to haemoglobin (*HBA*; *HBB*; *HBB-like* gene) and functions related to its synthesis and iron regulation. In mammals, mature erythrocytes are anucleated and, thus, do not produce new mRNA but retain mRNA, for the most part in the mature form^34^. This is indicated by the elevated relative presence of exonic sequences on total RNA and by the selective retention of mostly significant genes vs. nucleated erythrocyte progenitors. The majority of the remaining protein-coding mRNA found in erythrocytes codes for proteins relevant for proteins functional for their characteristics^34^, indicating that these cells are not just “bags of haemoglobin”. This argues in favour of a selective process of deletion of unnecessary mRNA during the maturation of reticulocytes and their differentiation into erythrocytes. The degree of retention of mature mRNA in porcine mature blood cells has not been documented. However, the gene for erythrocytic spectrin beta (*SPTB*), an important marker of young erythrocytes^35^, among the most upregulated genes (Supplementary Table 2) at weaning supports the idea of the presence of less mature erythrocytes at this phase as compared to the post-weaning phase. Nevertheless, there are also reticulocytes in the PB; reticulocytes are young, still immature erythrocytes and are 2–7% of the circulating red blood cells^36^; however, they do not replicate in circulation. Just-weaned pigs showed enriched gene sets related to haeme biosynthesis, cellular iron ion homeostasis, and erythrocyte development as compared to post-weaning pigs. This is the hallmark of a greater presence of cells of erythroid origin still under maturation. Erythropoietic activity is extraordinarily stimulated in newborn pigs^36,37^, and the still intense expression of genes could be related to the elevated presence of reticulocytes and/or of cells still retaining part of the functional mRNA. The greater percentage of reticulocyte cells in pigs was seen at 1 week or at 5–7 weeks of age^36^. The pigs in the present study were regularly given intramuscular iron at d3–d4 of age; however, successively the intense growth typical of late suckling and the insufficient iron content of the sow milk could have stimulated intense erythropoiesis. Iron deficiency and anaemia in young pigs at weaning has been seen in recent studies when a single dose of iron was given within 48 h from birth^38,39^. It is also possible that the lifespan of the reticulocytes increased in this phase, as in case of more intense release from the bone marrow^40^.

Gene sets related to protein translation, particularly to proteins targeting to membrane, and those related to the binding of extracellular ligands to an integrin on the cell surface, were also upregulated in the PB before weaning. The erythrocyte membrane has to be particularly resistant; however, it also has peculiar selective properties. This is obtained by a reorganisation during maturation from the reticulocyte condition^22^. Thus, these pathways are markers of the greater presence of erythroid cells under maturation in piglets.

Platelets are cellular fragments formed from the detached cytoplasm of large cells originating from bone marrow, megakaryocytes, and are anucleated, but can derive mRNA from the megakaryocytes, and are able to splice and post-transcriptionally manipulate it^41^. Platelets store a complex mix of molecules in granules which are necessary for coagulation, and the retraction and resolution of the coagulum. In the PB of just-weaned pigs, the gene set PLATELET_DEGRANULATION was enriched more than in post-weaned pigs. This could be due to the specific activation of this pathway or the enriched presence of the platelets. In effect, as reported in a companion paper submitted regarding the same set of pigs, the number of platelets was higher in the just-weaned pigs than in the post-weaning pigs, supporting the hypothesis that the enrichment of PLATELET_DEGRANULATION was due to their increased presence in the PB. Nevertheless, this set of genes was the most affected of all the others related to the platelets, indicating that it is the most sensible to variations. Data agree with the peaking of platelet cells observed at approximately 4 weeks of age of piglets^42^.

An interesting observation was the elevated expression of *PYY* and its upregulation in the PB from piglets at weaning (Ranking 41). *Peptide YY* encodes for a pre-protein which, depending on the site, pancreas or intestine, is cleaved to two different secreted peptides, related to controlling the meal and feed intake, a pancreatic peptide, or peptide YY. Thus, detecting these peptides in the blood is normal. However, no evidence was found in the research reported in the literature regarding a particular expression of *PYY* in the PB. In databases regarding gene expression in tissues, the PB is scarcely considered, and no evidence was found for *PYY*. The inspection of databases of trials reporting the porcine PB transcriptome supports the Authors’ observation^31,43^. It is difficult to explain that the increased expression obtained at weaning in the PB; this could indicate that *PYY* originates from the presence of reticulocytes or from young erythrocytes. Interestingly, PYY also has a vasoconstriction action; however, an association with the eventual production of PYY from the blood has never been proposed. It is thus tempting to hypothesise that the activity of this gene could be the result of a particular need of erythroid cells to control the vessel environment.

Overall, these aspects merit more studies owing to the increasing interest in the use of the PB transcriptome as a response parameter in trials on pigs, not limited to diagnostic purposes in pigs.

In piglets sampled 12 days after weaning, the degree of differential expression of single genes was more limited than in piglets at weaning. However, several enriched sets were related to DNA replication, chromatin remodelling, and chromosome segregation. This can be explained by a greater replicative pressure on the leukocytes as reticulocytes (and erythrocytes) have no replicative ability. As will be reported in a companion paper, the number of leukocytes was sharply increased post-weaning as compared to at weaning. The results of PB transcriptome evidence showed that the systemic pressure on immune system was still present after weaning. This is consistent with JPP data, at least for those concerned the MHC I stimulation.

### Effect of piglet age and weaning on jejunal microbiota

The microbiota of the content of the jejunum of piglets was dominated by the Firmicutes phylum which represented almost 97% of the total, with *Lactobacillus* and *Streptococcus* being the most abundant genus in accordance with previous studies^44,45^. Weaning significantly affected the bacterial population leading to an increase in bacterial richness; this has also been well reported in the literature^46^. A more diverse microbiota is considered to be a marker of a mature microbial community and is associated with functional redundancy, which contributes to a greater stability of the microbial ecosystem when it faces stressful situations, such as weaning, which can lead to dysbiotic conditions. A clear effect of weaning was also observed regarding the beta diversity, a parameter for which the samples clustered based on timepoint. The samples taken from piglets 12 days post-weaning had higher inter-variability as compared to piglets at weaning. The opposite pattern has been reported in other studies^46,47^ in which lactating piglets tended to have a higher inter-variability in relation to their individual intestinal maturation; then after weaning, there was a convergence towards a more stable microbial community. However, these studies mainly regarded faecal microbiota. Faeces represent almost the entire hindgut bacterial community which could potentially be less stable in the late suckling period in which the creep feed intake is variable between individuals, determining a variable adaptation of the microbiota^48^. Conversely, milk intake maintains a more stable microbiota in the small intestine^48^. On the contrary, in post-weaning pigs, it is also the small intestine which is impacted more by the relevant amount of solid feed, and the continuous influx of new bacteria from feed and from newly encountered piglets in the pen. In addition, the shift from a milk to a plant-based diet was accomplished by higher relative quantities of fibrolytic and/or short chain fatty acid producer bacterial groups, such as *Ruminococcus*, *Methanobrevibacter*, *Blautia* and *Subdoligranulum*, as also evidenced in several other studies^9,48^. These microorganisms are generally associated with the hindgut; however, fermentative activity also takes place in the distal small intestine, as indicated by classical studies regarding the quantification of the entity of the short chain fatty acids produced in the different digestive tracts^49^. The acute increase in *Collinsella aerofaciens* abundance is inside this shift as this microbe is also a producer of the acetate and lactate abundant in colon of humans^50^.

The dominant increased presence of *Streptococcus porcorum* was quite surprising as the primary isolation of this species in pigs^51^ was not from the intestine. It is facultatively anaerobic, and the recovery of this microbe in porcines isolates from lesions of pneumonia and arthritis^51^, and mesenteric lymph nodes and brain swab samples^52^ can render suspect its involvement under pathogenic conditions. However, no definitive conclusion regarding that has been reported, and the pigs in the present study were generally healthy.

In conclusion, JPPs sampled after weaning presented a general upregulation of the genes involved in the activation of class I MHC response and IFN-γ response as compared to just-weaned pigs. The intensity of this upregulation was only marginally associated with the variation found in the corresponding gut microbiota which, conversely, differs from that seen in just-weaned pigs. The transcriptome of these pigs presented an interesting upregulation of the genes attributable to reticulocytes and erythrocytes under maturation. Several of these genes could be interesting for testing iron deficiency in piglets.

## Material and methods

On the day of weaning (26 days of age), 32 piglets ((Large White × Landrace × Duroc crossbred) from 8 l (average live weight 7.18 kg, s.d. 0.77) were obtained from a commercial farm, and were assigned to two different times of slaughtering (the groups were balanced for the litter of provenience and live weight): weaning time or 12 days after weaning. The piglets were still with their sows when they were collected and were then immediately transported all together for 1 h and half in a van to the experimental facility of the University of Bologna. There, the first group (nine females, seven males) was immediately slaughtered while the second group (eight females, eight males) was raised for twelve days with the same pre-starter feed used during the suckling period (Supplementary Table 5) and were finally slaughtered at 12 days (average live weight 9.76 kg, s.d. 0.57). After weaning, the pigs were reared individually and were penned inside a weaning room at pre-controlled temperatures and ventilation. Feed and water were freely available. On the morning of the day of sacrifice, they received half the dose of feed which had been given in the second meal of the previous day and access to the feed was closed an hour and half before the sacrifice.

After sedation by anaesthesia with tiletamine (15 mg/kg), the pigs were sacrificed with an intracardiac injection of a solution mixture of embutramide/mebezonium iodide/tetracaine hydrochloride (Tanax^©^, MSD Animal Health srl, Segrate, Italy; 0.5 mL/kg). Per each pig, a sample of PB was obtained by venipuncture on the vena cava before anaesthesia for mRNA sequencing, and a sample of JPPs was later collected from the jejunum, at approximately the 66% point of the small intestine length. The JC was also obtained at the same position, from a section of about 20 cm. The PB was collected using Tempus™ blood RNA tubes (Thermo Scientific, Waltham, MA, USA) while the JPPs and JC were collected in sterile tubes and were immediately frozen in liquid nitrogen. All the samples were then stored at − 80 °C until processed.

### Pig mRNA extraction and sequencing

Total PB RNA was extracted from the Tempus™ tubes using the Tempus™ Spin RNA Isolation Kit (Thermo Scientific, Waltham, MA, USA) and from the JPP samples using the GeneJET RNA Purification Kit (Thermo Scientific, Waltham, MA, USA), following the manufacturer’s instructions. Contaminating DNA was removed by DNase treatment using the TURBO DNA-free™ DNA Removal Kit (Thermo Scientific, Waltham, MA, USA) following the recommended protocol. The RNA quantity and quality were evaluated using a Nanodrop ND 1000 spectrophotometer (Nanodrop Technologies Inc., Wilmington, DE, USA) and agarose gel electrophoresis, respectively. An Agilent Bioanalyzer 2100 (Agilent Technologies, Santa Clara, CA, USA) was used for testing RNA integrity. Libraries were prepared using the TruSeq Stranded mRNA Sample Preparation kit and were sequenced using the Illumina MiSeq system 2 × 100 bp with 2 × 20 million sequencing depth.

### Differential expression analyses of RNA-Seq data

The reads were quality controlled using the FastQC tool (v.0.11.9), filtered with Trimmomatic (v.0.36)^53^ by trimming leading and trailing bases with a Phred score less than 2 and dropping reads shorter than 15 bases long. Those with an average Phred score per base of less than 15. Salmon (v.0.14.1)^54^ were used to align sequences to the National Center for Biotechnology information (NCBI) *Sus scrofa* v11.1 reference transcriptome and are publicly available at the NCBI Sequence Read Archive under accession number SUB8684880.

An initial exploratory analysis of the expression profile was conducted using an MDS plot, based on count data normalised using variance stabilised transformation which was based on the count data of all the genes. In this plot, the samples were positioned according to the statistical distance of their expression profiles. The differential effect of the two different time points on single gene expression was carried out in R (3.6.2) using a DESeq2 package (v.1.26)^55^. The genes were considered to be differentially expressed when the FDR was < 0.05.

### Functional enrichment analysis

An exploratory analysis was conducted using GSEA software to evidence the enriched gene sets having a common biological function, depending on the time of sampling. The GSEA analysis was based on the C5 Biological Process collection of Gene Ontology^56^ (MSigDB, Broad Institute, and UC San Diego). Gene sets with an FDR (q value) ≤ 0.05 were considered to be significantly enriched. The overlap and connections between the resulting different gene sets for the different times were produced by the EnrichmentMap Plugin (http://baderlab.org/Software/EnrichmentMap) for Cytoscape 3.8^57^, considering a q value of FDR < 0.05 or < 0.01, depending on how it was later specified. The nodes were joined if the overlap coefficient was ≥ 0.375.

### Microbiota

The bacterial DNA extraction from the JC was carried out using a HostZERO Microbial DNA Kit (Zymo Research, California, USA) following the manufacturer’s instructions. The DNA concentration and purity (absorbance ratio 260/280 and 260/230, respectively) of the DNA isolated were checked using spectrophotometry on NanoDrop (Fisher Scientific, 13 Schwerte, Germany). The V3-V4 region of the 16S rRNA gene (~ 460 bp) was amplified, amplicons were produced using the universal primers Pro341F: 5′-TCGTCGGCAGCGTCAGATGTGTATAAGAGACAGCCTACGGGNBGCASCAG-3′ and Pro805R: 5′GTCTCGTGGGCTCGGAGATGTGTATAAGAGACAGGACTACNVGGGTATCTAATCC-3′ ^58^ using Platinum™ Taq DNA Polymerase High Fidelity (Termo Fisher Scientific, Italy) and sequenced using the Illumina MisSeq platform 300 × 2 bp. The libraries were prepared using the standard protocol for MiSeq Reagent Kit V3 and were sequenced on the MiSeq platform (Illumina Inc., San Diego, CA, USA). For the bioinformatics analysis, the DADA2 pipeline was utilised^59^, using the Silva database (version 138)^60^ as reference for the taxonomic assignment. The raw reads obtained from the 16s sequencing are publicly available at the NCBI Sequence Read Archive under accession number SUB9499525.

The statistical analyses regarding Alpha diversity, Beta diversity and taxonomic composition were carried out with R v3.6. The data were normalised using the variance stabilising transformation function provided by the DESeq2 package^55^. A simple linear model assessed the effect of time on Alpha diversity. For the Beta diversity, a dissimilarity matrix using Euclidian distance was constructed; the results were plotted using an MDS plot, and a PERMANOVA was subsequently carried out to test for any correlation between community composition and age, with 10,000 permutations. The differential abundance analysis was carried out using the DESeq2 package, and the data were aggregated at the Genus level.

### Clustering of weaned pigs for the MHC class I in JPPs and the effect on jejunal microbiota

After examination of the JPP transcriptome data (cfr. Results section), the pigs sampled at 12 days post-weaning were clustered into two groups based on the gene expression of a total of 44 genes related to the MHC class I antigen and to the response to IFN-γ production (Supplementary Table 6). In particular, they were those in the enriched core of the gene sets ANTIGEN_PROCESSING_AND_PRESENTATION_OF_ENDOGENOUS_ANTIGEN; INTERFERON_GAMMA_RESPONSE and of the MULTIVESICULAR_BODY_SORTING_PATHWAY. The transcriptome values were standardised using Z-score scaling so that, within each gene, mean = 0 and standard deviation = 1. The standardised sets of gene values for each pig were transformed using principal component analysis (PCA), with Proc PRINCOMP in SAS, to reduce the number of variables for additional testing. The distribution of samples was visualised on the score plot using Proc GPLOT of SAS. The components from the PCA which explained 85% of the total gene transcript variability and had eigenvalues for components > 0.80 (Supplementary Fig. 3) were used to create similarity clusters based on partitional clustering methods (Proc FASTCLUS in SAS). The resulting attribution cluster of each pig was used as a dependent variable to test the effect on the microbiota of the JC of the same pig. Alpha and beta diversity were analysed using a linear or PERMANOVA model, respectively, and using the Cluster attribution of each subject as a covariate. Taxonomic differences were tested using DESeq2, aggregating the data at the genus level.

### Ethics approval

The procedures complied with Italian law pertaining to experimental animals and were approved by the Ethic-Scientific Committee for Experiments on Animals of the University of Bologna, Italy and by the Italian Ministry of Health (Prot. N. 675-3/96/2018). The study was carried out in compliance with ARRIVE guidelines.

## Supplementary Information

Supplementary Information.

## Data Availability

Transcriptome and microbiome data are publicly available at the NCBI Sequence Read Archive under accession numbers SUB8684880 and SUB9499525, respectively.
