# Supplementary material for: Weaning differentially affects the maturation of piglet peripheral blood and jejunal Peyer’s patches
Source: Sci Rep. 2022 Jan 31;12:1604. doi: 10.1038/s41598-022-05707-9 (PMC8803882; doi:10.1038/s41598-022-05707-9)

Supplementary Information file

Weaning differentially affects the maturation of piglet peripheral blood and jejunal Peyer's patches

Federico Correa, Diana Luise, Paolo Bosi, Paolo Trevisi*

Supplementary table 1 List of genes with statistically significant differential expression in jejunal Payer's patches collected from pigs at weaning or 12 d post-weaning

Gene	baseMean	log2FoldChange	lfcSE	padj
At weaning				
MELTF	139.6	-3.89	0.76	0.000291
LOC100523635	2.7	-3.46	1.02	0.04639
LOC110256816	48.6	-3.44	0.77	0.002658
RTP3	14.7	-3.35	0.92	0.026481
FGF19	25.2	-3.29	0.89	0.025189
LOC106510103	4.4	-3.03	0.90	0.047563
LOC110258600	6.8	-2.99	0.84	0.034228
LOC106509501	4.0	-2.88	0.76	0.020469
SULT1E1	52.0	-2.84	0.63	0.002658
CGREF1	202.0	-2.58	0.43	7.17E-06
LOC110256001	4.1	-2.53	0.71	0.03356
APOC2	13.9	-2.32	0.48	0.000715
TUBB1	10.2	-2.30	0.53	0.003374
KLHL10	4.4	-2.25	0.57	0.012432
LOC106505545	9.8	-2.24	0.59	0.018636
LOC102168060	25.1	-2.21	0.49	0.002658
ATOH7	195.9	-2.19	0.52	0.006193
LOC110255329	6.2	-1.98	0.57	0.043038
CLIC6	101.3	-1.96	0.42	0.001758
RN5-8S	4295.3	-1.96	0.44	0.003017
LOC110259943	6.9	-1.84	0.54	0.047827
LOC110261483	25.7	-1.82	0.51	0.03207
LOC102161678	5.6	-1.81	0.51	0.035375
DNAH7	11.2	-1.77	0.43	0.008822
GABRQ	3.8	-1.69	0.50	0.049997
GPD1	592.4	-1.66	0.41	0.008438
ITGAD	359.3	-1.66	0.41	0.009097
LOC106505804	348.5	-1.65	0.43	0.017236
SCNN1A	46.9	-1.64	0.43	0.017236
LOC102166468	8.3	-1.63	0.46	0.035006
VPREB1	377.0	-1.61	0.46	0.037152
RAB30	803.6	-1.55	0.38	0.008832
LOC102159548	7.6	-1.53	0.45	0.045939
CLEC20A	43.6	-1.51	0.40	0.020425
LOC110261134	15.9	-1.49	0.42	0.033231
SHISA8	11.9	-1.49	0.43	0.043465
HAP1	13.3	-1.47	0.39	0.021296
RIMBP2	32.9	-1.41	0.30	0.001165
LOC110256168	3.4	-1.41	0.41	0.046333
NUPR1	787.0	-1.35	0.31	0.003903
EBF4	88.6	-1.33	0.31	0.004158
PIWIL2	63.9	-1.31	0.34	0.018459
LOC102160003	169.1	-1.30	0.33	0.015358
LOC106508121	10.1	-1.28	0.36	0.035006

PDK4	747.1	-1.28	0.37	0.039589
LOC102165634	1790.4	-1.28	0.38	0.046887
LOC110261321	59.9	-1.25	0.31	0.011266
ZBTB16	176.9	-1.24	0.31	0.012432
HOMER2	82.5	-1.23	0.36	0.047844
HSPA1L	200.8	-1.20	0.31	0.015706
EPS8L1	12.5	-1.18	0.35	0.048139
MET	175.2	-1.17	0.33	0.033231
GPIHBP1	15.9	-1.16	0.29	0.009354
LOC100510887	721.4	-1.15	0.32	0.028942
LOC110261661	15.6	-1.14	0.28	0.009097
ADAMTSL4	231.1	-1.12	0.25	0.002658
SNX32	38.8	-1.08	0.30	0.032381
VSIG4	88.3	-1.07	0.27	0.012125
SIK1	173.5	-1.06	0.27	0.012757
CD163	923.8	-1.05	0.31	0.044125
FUT7	20.2	-1.05	0.31	0.044905
LRRC10B	50.9	-1.02	0.26	0.010915
FMO1	80.1	-0.98	0.26	0.019598
CPT1A	2377.3	-0.98	0.21	0.001913
SLC2A8	76.1	-0.98	0.29	0.047563
CCDC146	8.1	-0.97	0.29	0.047844
LOC110261315	168.4	-0.95	0.21	0.00235
LOC106507497	12.8	-0.92	0.27	0.042923
PEG3	133.8	-0.92	0.24	0.018636
TTPA	71.4	-0.90	0.25	0.035884
CD302	247.6	-0.86	0.18	0.001372
LIPE	386.6	-0.85	0.21	0.00995
C6H19orf68	21.7	-0.84	0.24	0.033191
LOC110255260	49.5	-0.84	0.18	0.001274
LOC100525078	79.1	-0.84	0.19	0.002707
LOC110261322	71.9	-0.84	0.20	0.00535
LPGAT1	2746.3	-0.84	0.25	0.048665
BTG2	1180.8	-0.81	0.22	0.023411
PIMREG	101.3	-0.80	0.24	0.049676
LOC106506219	19.5	-0.79	0.21	0.020919
LOC100513863	1163.1	-0.79	0.23	0.047435
LOC110262106	18.1	-0.79	0.22	0.035884
APOLD1	61.6	-0.76	0.21	0.033827
BEX1	75.1	-0.75	0.22	0.047844
CCDC92	32.3	-0.75	0.21	0.033358
SERPINI1	496.7	-0.74	0.18	0.011266
COQ10A	54.0	-0.73	0.16	0.002658
LONRF3	124.8	-0.73	0.20	0.023411
RRAGD	39.9	-0.72	0.19	0.0222
ITIH3	57.8	-0.72	0.19	0.020469
GGT7	319.2	-0.72	0.19	0.019591
PHACTR1	425.1	-0.71	0.21	0.042923
LRRC3	32.4	-0.69	0.19	0.022715

CD300LG	74.2	-0.69	0.15	0.002658
LOC100625694	48.3	-0.69	0.17	0.008152
LOC110255314	35.7	-0.69	0.20	0.049889
CERS4	261.1	-0.68	0.20	0.043465
FKBP5	2079.0	-0.67	0.16	0.005887
PTPMT1	294.4	-0.67	0.18	0.022715
LOC100513741	211.1	-0.66	0.17	0.018636
GEMIN7	421.0	-0.65	0.18	0.035937
TCEAL8	482.4	-0.63	0.17	0.028569
CXXC5	378.2	-0.61	0.13	0.001508
LOC100624963	45.9	-0.58	0.15	0.011605
HMOX1	422.6	-0.58	0.17	0.043696
PGBD1	181.4	-0.58	0.17	0.044433
DCLK2	41.4	-0.57	0.16	0.039827
INAFM2	375.3	-0.57	0.16	0.039409
SLC15A3	412.3	-0.56	0.16	0.042923
MTR	273.2	-0.56	0.16	0.04639
C6H1orf174	190.6	-0.55	0.14	0.020469
ARID3A	613.3	-0.53	0.15	0.042848
TCEANC	276.9	-0.52	0.15	0.047452
IFT27	109.1	-0.46	0.13	0.037331
ERO1B	325.5	-0.45	0.13	0.049226
TMEM263	1272.8	-0.43	0.10	0.007551
C8H4orf46	120.2	-0.42	0.11	0.017388
FAM53C	506.3	-0.41	0.11	0.024834
OAZ2	579.4	-0.40	0.11	0.026481
TBC1D7	500.0	-0.40	0.11	0.025422
ZNF287	129.9	-0.39	0.11	0.041534
TTPAL	480.6	-0.37	0.11	0.047563
LOC100523964	140.1	-0.37	0.11	0.047563
KLHDC3	675.2	-0.36	0.10	0.035006
USP10	1607.2	-0.33	0.10	0.047435
GDI2	7582.1	-0.26	0.07	0.017388
Post-weaning				
PCSK9	107.6	3.46	0.39	1.35E-14
LOC110256411	5	3.23	0.73	0.003079
LOC106507448	12.2	2.62	0.64	0.008349
CYP1A1	60.4	2.54	0.42	4.70E-06
ALOXE3	3.1	2.5	0.61	0.008822
LOC110260234	5.4	2.43	0.39	1.98E-06
WT1	2.9	2.43	0.7	0.042575
PLB1	1064.5	2.38	0.55	0.004158
UBD	8464.8	2.22	0.38	1.54E-05
LOC102162420	130	2.22	0.4	4.24E-05
SLC22A13	269.6	2.17	0.54	0.010231
LOC100624460	5.9	2.08	0.54	0.016755
TTLL8	4.6	2.03	0.52	0.014506
LOC106505337	28.1	1.97	0.48	0.009097
MGAM	28817.3	1.91	0.4	0.001087

LOC106507518	298.9	1.91	0.5	0.019598
GZMB	2277.7	1.81	0.34	0.000112
LOC100154312	12.2	1.8	0.35	0.000203
NOS2	2204.2	1.8	0.43	0.006136
LOC106507519	332.7	1.76	0.43	0.009097
LOC100515902 (SLA class I histocompatibility antigen, A-11 alpha chain)	936	1.74	0.38	0.001913
AHRR	60.4	1.73	0.33	0.00015
LOC106506487	9.9	1.73	0.5	0.042923
NEURL3	287.5	1.73	0.29	8.48E-06
PLA2G3	35	1.69	0.31	4.85E-05
LOC102161780	37.6	1.68	0.31	9.27E-05
KLRD1	52.5	1.68	0.47	0.03411
NOX1	23.6	1.66	0.45	0.02344
CD96	169.8	1.65	0.27	3.50E-06
BPI	384.1	1.59	0.44	0.031279
LOC100520680	38	1.58	0.38	0.008152
GAPT	37.9	1.57	0.37	0.004699
SI	31895.3	1.56	0.38	0.008868
LOC100523492	676.3	1.53	0.35	0.003374
CD244	217.1	1.53	0.26	1.20E-05
SULF2	334.5	1.52	0.24	1.98E-06
CCL24	570.7	1.52	0.38	0.01092
NCR2	6.1	1.5	0.31	0.000663
CD200R1L	121	1.49	0.4	0.021341
SLC15A1	6232.5	1.48	0.37	0.009976
LYZ	32025.2	1.47	0.4	0.02892
SLC1A1	6371.7	1.45	0.41	0.033231
LOC102165774	18.6	1.44	0.41	0.039409
GZMA	2685.3	1.43	0.32	0.002631
CD274	384.2	1.42	0.39	0.026481
LOC102167681	8	1.42	0.31	0.00241
GNLY	633.7	1.42	0.32	0.002707
GBP2	6900	1.42	0.34	0.005678
COLCA1	8.2	1.41	0.41	0.046333
KLRK1	198.1	1.4	0.3	0.001758
CHRD	88	1.4	0.32	0.004118
LOC396781	12360.7	1.39	0.41	0.04639
THPO	13.7	1.36	0.37	0.026481
PIK3C2G	41.9	1.32	0.37	0.034228
MLC1	96.9	1.29	0.37	0.042848
TMEM61	107.3	1.27	0.36	0.033231
CD3D	265.8	1.27	0.22	1.67E-05
ASS1	8825.3	1.27	0.35	0.030329
LPAR3	15.1	1.26	0.27	0.001508
LOC100523668	4587.5	1.25	0.28	0.002893
LOC110260348	1666.9	1.24	0.31	0.011
SLC2A5	913.1	1.24	0.36	0.042923

SEMA6D	1551.1	1.23	0.25	0.000647
SGIP1	651.7	1.23	0.31	0.012146
SCD	1133.7	1.22	0.22	3.55E-05
FASLG	18.6	1.22	0.29	0.007663
LOC102160313	1138.7	1.21	0.27	0.003079
XCL1	126.4	1.21	0.23	0.000207
LOC100736962	3267.5	1.16	0.34	0.049889
LOC100520273	12986	1.15	0.34	0.046333
MST1	114.7	1.14	0.26	0.003079
CD40LG	29.6	1.13	0.28	0.010807
LOC100519314	91.7	1.12	0.29	0.014501
CD3G	850.8	1.11	0.2	2.40E-05
LOC110255596	17.2	1.1	0.29	0.018421
LOC106509632	13.4	1.08	0.3	0.033231
CD3E	1551.4	1.07	0.19	4.58E-05
LOC106510284	2155.7	1.06	0.29	0.0222
LOC110255214	1849.4	1.05	0.29	0.027193
LOC100620407	27.1	1.03	0.26	0.010047
CD226	108	1.02	0.24	0.004699
SYT2	74.5	1.02	0.25	0.010286
LOC110255290	23.4	1.02	0.3	0.04639
LOC106510360	31.7	1	0.28	0.033231
BATF2	943	1	0.27	0.024693
PIGR	10477.5	1	0.3	0.049676
IL15	178.2	1	0.27	0.026481
LOC110255361	974.2	0.99	0.26	0.018636
TNS4	409.2	0.99	0.21	0.001758
TOX2	53.6	0.99	0.28	0.03207
RNF180	497.4	0.99	0.28	0.033231
TIAM2	127.8	0.98	0.2	0.000647
PRR13	34098.3	0.98	0.29	0.046333
ITGAE	758.7	0.98	0.19	0.00015
LOC100155579	5076.1	0.98	0.28	0.037048
TNFSF10	6938.1	0.97	0.27	0.033231
TLDC2	1920.7	0.97	0.28	0.039594
LOC100620198	354.6	0.96	0.2	0.001092
LCAT	757.6	0.96	0.22	0.003358
SHF	48.3	0.96	0.22	0.003079
C4BPA	14835.7	0.95	0.24	0.015521
CD101	94	0.95	0.27	0.033231
GPR55	143.8	0.92	0.2	0.001926
IL15RA	226	0.9	0.25	0.025884
TAP1	8476.7	0.89	0.19	0.001913
DAPK2	78.9	0.88	0.2	0.003079
CD8A	587.7	0.88	0.21	0.006536
SLC6A20	1147.7	0.88	0.25	0.035006
THEMIS	76.4	0.87	0.23	0.01833
LOC100154873	48.2	0.86	0.23	0.020048
CRTAM	79.1	0.86	0.23	0.022294

CD38	386.9	0.86	0.24	0.03356
IRF1	5551.8	0.86	0.21	0.010231
PTPN22	232.9	0.86	0.23	0.018459
PRF1	149.9	0.84	0.23	0.025189
CCL5	2110	0.84	0.25	0.049676
LOC100522011	1007.4	0.84	0.23	0.027127
GSDMB	5098.4	0.82	0.24	0.049392
SOWAHD	76.7	0.81	0.2	0.012757
TRERF1	134.4	0.81	0.24	0.04639
MGST1	8506.6	0.81	0.24	0.049676
TIGIT	257.9	0.8	0.24	0.049401
NPAS2	231.2	0.8	0.22	0.023411
LOC100523440	176.7	0.78	0.22	0.042575
WARS	2498.6	0.78	0.23	0.047563
LOC110255499	26.3	0.78	0.19	0.009097
CD6	249.1	0.77	0.2	0.016899
CD2	836	0.77	0.18	0.00535
PSMB9	4165.4	0.76	0.16	0.001758
IL2RB	1040.1	0.75	0.15	0.000291
NEURL1B	886.5	0.74	0.21	0.035868
SLA2	451	0.72	0.16	0.003358
GPR68	55.9	0.71	0.17	0.006965
APOBR	289.5	0.71	0.19	0.028059
LOC100739325	313.6	0.7	0.19	0.025911
LOC100626505	652.4	0.7	0.18	0.016294
APLNR	135.2	0.7	0.21	0.046333
PRKCH	297.9	0.7	0.15	0.001508
CRACR2A	126.6	0.69	0.18	0.020745
EPHB6	1259.7	0.69	0.19	0.023411
SH2D1B	71.5	0.68	0.19	0.025884
NKG7	473.2	0.63	0.19	0.048139
LAP3	6558.5	0.61	0.17	0.038212
ME2	2771.6	0.61	0.17	0.041324
C2H5orf30	534	0.61	0.18	0.047844
ADGRG1	759.1	0.6	0.16	0.018636
PSMB10	2688.6	0.6	0.17	0.042923
RNF19B	1498.8	0.59	0.16	0.020478
TAP2	3644.2	0.58	0.17	0.043694
SLC20A2	183.6	0.58	0.16	0.026029
TOX	364.3	0.56	0.13	0.006256
NUB1	2140.4	0.5	0.15	0.049676
JAML	1170.4	0.48	0.13	0.018459
KYNU	767	0.47	0.14	0.049676
SPATA13	638.4	0.47	0.11	0.003079
CITED2	789.8	0.47	0.13	0.030754
DDX59	412	0.46	0.12	0.02154
DENND3	435.8	0.46	0.12	0.0222
OSBPL11	439.4	0.46	0.12	0.01833
DLG2	119.5	0.45	0.13	0.042848

METTL4	246.7	0.43	0.12	0.03207
CNOT2	1816	0.4	0.12	0.049676
WWOX	149.7	0.35	0.1	0.035006
TAPBP	5336.3	0.34	0.1	0.044905

Supplementary table 2 List of gene with statistically significant differential expression in peripheral blood sampled from pigs at weaning or 12 d post-weaning

**Gene**	**baseMean**	**log2FoldChange**	**lfcSE**	**padj**
At weaning				
PI15	2.4	-4.52	1.03	0.000533
GFY	3.5	-4.13	0.95	0.000628
PRR9	1.6	-3.96	1.32	0.035421
COL5A3	1.6	-3.80	1.18	0.020112
DLX2	2.7	-3.73	0.97	0.003628
CCDC42	443.0	-3.47	0.60	2.01E-06
ACTA1	3.8	-3.41	1.13	0.033307
TMCC2	7370.1	-3.40	0.56	5.99E-07
F2RL2	1.9	-3.33	1.12	0.038851
ACHE	47.0	-3.25	0.50	1.32E-07
UGGT2	50.9	-3.19	0.55	1.63E-06
LOC102158609	7.6	-3.17	1.10	0.046674
SPTB	560.6	-3.10	0.54	2.01E-06
HENMT1	4.7	-3.08	0.81	0.004105
COL4A5	1.7	-3.07	0.96	0.021066
FECH	16098.7	-3.01	0.53	2.56E-06
TNNC2	2.1	-2.98	1.01	0.040317
SPTA1	379.2	-2.93	0.62	0.00015
ANKLE1	707.3	-2.91	0.48	4.52E-07
MMP8	671.3	-2.91	0.64	0.000301
STK32A	5.1	-2.91	0.90	0.019919
ELN	123.7	-2.88	0.50	1.83E-06
ADD2	687.6	-2.87	0.50	2.47E-06
MIOX	114.4	-2.85	0.59	9.82E-05
TSPO2	106.3	-2.85	0.48	1.27E-06
TSKU	169.1	-2.83	0.51	3.76E-06
FGF12	6.6	-2.83	0.74	0.003688
LOC106505983	234.6	-2.82	0.45	2.21E-07
ACTN2	2.5	-2.80	0.94	0.036681
EPB42	5094.1	-2.79	0.47	1.11E-06
LOC110256048	1.8	-2.79	0.79	0.00841
CELF3	22.7	-2.79	0.47	9.42E-07
BCL2L1	7194.4	-2.77	0.44	2.53E-07
PDLIM3	1.4	-2.76	0.80	0.011585
DYNLRB2	96.8	-2.76	0.49	3.39E-06
LMCD1	1.6	-2.73	0.88	0.028101
WFDC2	66.0	-2.68	0.61	0.000467
REEP1	1812.5	-2.68	0.50	1.32E-05

RHCE	1404.5	-2.67	0.47	2.02E-06
PDGFRL	1.2	-2.67	0.88	0.032968
NRN1L	25.3	-2.66	0.41	1.01E-07
PDK4	6.0	-2.66	0.63	0.000994
FAM50B	3.1	-2.65	0.71	0.004685
SRPX	1.5	-2.65	0.77	0.012056
PYY	16262.2	-2.64	0.53	4.63E-05
TRIM58	1092.5	-2.62	0.46	3.01E-06
DPT	4.9	-2.62	0.73	0.007988
CCNDBP1	9283.6	-2.60	0.42	3.03E-07
ITGA11	1.2	-2.60	0.81	0.021443
CMAS	5156.0	-2.60	0.42	2.66E-07
SH3RF2	14.0	-2.60	0.57	0.000268
CKM	2.9	-2.56	0.75	0.011986
LOC110260291	4.3	-2.54	0.56	0.000292
EPOR	10.7	-2.54	0.54	0.000173
MARCH3	466.3	-2.53	0.47	7.85E-06
LOC106506844	21.9	-2.53	0.68	0.004932
CTRB2	27.4	-2.52	0.75	0.013896
PHOSPHO1	794.1	-2.51	0.46	6.09E-06
SYN3	67.8	-2.50	0.51	6.46E-05
CRHR2	59.3	-2.50	0.55	0.000266
CST6	28.1	-2.50	0.36	2.47E-08
ENDOU	2.6	-2.48	0.73	0.013387
RAD23A	4676.3	-2.45	0.37	8.83E-08
FAM213A	6241.6	-2.45	0.45	6.67E-06
KCNN3	58.5	-2.44	0.34	7.67E-09
LOC110259924	1715.2	-2.44	0.52	0.000139
KEL	840.6	-2.43	0.36	4.37E-08
ARG1	19315.2	-2.41	0.41	1.34E-06
NCOA4	37513.3	-2.41	0.46	1.33E-05
CREB3L3	492.3	-2.40	0.37	9.84E-08
IGFBP6	2.5	-2.40	0.64	0.004549
CDKL4	2.1	-2.39	0.75	0.021699
LOC110260147	108.2	-2.39	0.40	9.61E-07
YPEL4	252.3	-2.38	0.44	7.74E-06
LOC100523684	793.0	-2.38	0.39	6.73E-07
LOC110255596	125.1	-2.37	0.46	2.22E-05
PLXDC1	2.4	-2.37	0.76	0.02756
ADIRF	5.1	-2.36	0.45	1.81E-05
TRPV5	10.1	-2.36	0.55	0.000643
FXYD3	337.6	-2.36	0.49	0.000101
LOC102161036	6.5	-2.36	0.65	0.00652
LOC100524479	19.0	-2.35	0.47	3.71E-05
HP	434.1	-2.35	0.48	8.21E-05
SEMA3G	2.9	-2.34	0.65	0.006746
FAM46C	14726.0	-2.34	0.41	3.01E-06
CFAP58	3.4	-2.33	0.63	0.005635
TMEM225B	2535.8	-2.32	0.44	1.22E-05

SOX6	564.7	-2.31	0.45	2.14E-05
BPGM	3805.8	-2.30	0.32	2.89E-09
RUNDC3A	237.7	-2.28	0.40	3.07E-06
LOC100153915	494.1	-2.27	0.49	0.000185
HBQ1	69.4	-2.26	0.42	1.12E-05
SLC25A21	106.5	-2.25	0.41	4.54E-06
CYP3A22	78.3	-2.24	0.62	0.006519
LOC110261483	8.6	-2.24	0.40	4.78E-06
RHAG	667.4	-2.23	0.44	3.35E-05
NETO2	108.2	-2.22	0.39	2.01E-06
EPHB2	5.2	-2.21	0.57	0.002964
EPB41	46638.1	-2.21	0.43	3.07E-05
UBE2O	12053.9	-2.20	0.43	2.42E-05
LOC106506368	1.2	-2.20	0.73	0.035355
ZNHIT1	470.4	-2.20	0.38	1.83E-06
LOC102164789	25.9	-2.20	0.28	9.97E-11
LTF	2164.9	-2.20	0.44	3.73E-05
BGN	11.9	-2.19	0.72	0.031652
CSPG4	4.1	-2.19	0.75	0.041286
HMBS	1848.3	-2.19	0.38	1.49E-06
ALAD	1027.6	-2.19	0.38	2.47E-06
R3HDM4	31711.9	-2.18	0.46	0.000135
KLF1	23.8	-2.18	0.40	6.09E-06
ARL4A	484.9	-2.17	0.36	6.17E-07
LOC110260264	6.3	-2.16	0.56	0.003246
PRDX2	9808.5	-2.15	0.35	3.95E-07
SYNC	82.8	-2.15	0.41	1.80E-05
LGI4	175.3	-2.15	0.41	1.36E-05
ART5	79.5	-2.14	0.42	2.69E-05
ASPDH	4.5	-2.14	0.55	0.003203
LOC110256053	2631.0	-2.13	0.37	1.81E-06
IGF2	20.3	-2.13	0.46	0.000197
LAMA4	2.3	-2.13	0.65	0.018864
CXCL8	126.1	-2.13	0.57	0.004472
TNNI1	1094.1	-2.12	0.49	0.000616
CGNL1	3.4	-2.12	0.53	0.00208
ANTXRL	34.7	-2.11	0.43	6.20E-05
LOC100737180	1.5	-2.11	0.70	0.036097
ZNF469	1.2	-2.11	0.69	0.031979
STRADB	16956.5	-2.10	0.43	8.81E-05
LOC100154873	2026.3	-2.10	0.50	0.000936
FIS1	2774.8	-2.10	0.36	1.95E-06
HEMGN	5624.7	-2.09	0.44	0.000135
SNCG	45697.4	-2.09	0.43	9.82E-05
LOC110261665	1.3	-2.08	0.61	0.013124
GATA1	380.1	-2.08	0.35	6.73E-07
ISCA1	6990.1	-2.08	0.32	8.83E-08
EN1	28.1	-2.07	0.42	5.66E-05
HBZ	282.1	-2.07	0.39	1.59E-05

LOC110259374	24682.8	-2.07	0.34	3.99E-07
AQP1	1526.9	-2.07	0.36	2.01E-06
MXI1	4755.8	-2.06	0.33	2.93E-07
TMEM86B	1067.6	-2.06	0.38	5.66E-06
LOC110255525	7.1	-2.06	0.60	0.0111
CRISP3	81.1	-2.06	0.44	0.000181
ATP4B	191.3	-2.06	0.49	0.001118
LOC106508201	15.7	-2.06	0.50	0.001427
GYPC	1621.6	-2.05	0.33	4.37E-07
ACKR2	3.0	-2.05	0.59	0.010098
CRAT	12630.6	-2.03	0.34	1.22E-06
PODXL2	58.2	-2.03	0.35	1.41E-06
RANBP10	8751.4	-2.02	0.39	1.56E-05
LOC100625498	19.3	-2.02	0.39	1.90E-05
JOSD2	233.1	-2.02	0.39	2.18E-05
MYOZ1	6.3	-2.02	0.59	0.011506
TGM3	14803.9	-2.02	0.44	0.000273
CYB5A	2970.7	-2.01	0.33	4.35E-07
LOC106506763	7754.9	-2.01	0.33	4.44E-07
TSPAN31	1988.6	-2.01	0.36	3.44E-06
ERMAP	171.6	-2.00	0.37	1.12E-05
SLC14A1	26.2	-1.99	0.46	0.000657
MARVELD3	1.5	-1.99	0.68	0.039792
HAGH	4204.7	-1.99	0.38	1.85E-05
LOC100523213	32.0	-1.99	0.38	1.80E-05
SLC25A39	38293.7	-1.98	0.45	0.000446
LOC110259877	2.3	-1.98	0.61	0.020983
AP3B2	6.7	-1.97	0.51	0.003119
ASB10	3.7	-1.97	0.59	0.015374
LOC106505355	69.4	-1.95	0.43	0.000344
TMEM200B	9.6	-1.95	0.50	0.002864
IL1R2	2294.2	-1.94	0.45	0.000643
BLVRB	1972.3	-1.94	0.35	3.44E-06
ART4	22.4	-1.94	0.42	0.000202
UBALD1	654.9	-1.93	0.36	1.00E-05
TFDP2	9252.7	-1.93	0.35	3.82E-06
LOC100512420	14.6	-1.93	0.44	0.000576
LOC110260186	5.2	-1.92	0.45	0.000841
HBA	5781004.5	-1.92	0.43	0.000415
FTL	419945.1	-1.92	0.44	0.000576
TGM7	7.5	-1.92	0.38	3.25E-05
SLC4A1	3448.2	-1.92	0.44	0.000581
GYPA	5074.4	-1.91	0.44	0.000607
TRIM10	286.7	-1.91	0.43	0.000468
LOC110260892	78.3	-1.90	0.35	8.67E-06
LOC110260441	129.6	-1.90	0.31	3.74E-07
LOC102163428	33.3	-1.89	0.34	4.02E-06
ALAS2	458294.9	-1.89	0.41	0.000268
LOC100519918	18.5	-1.89	0.60	0.024952

DMTN	15359.9	-1.89	0.42	0.000288
MFAP5	6.1	-1.89	0.61	0.027959
RGCC	1301.2	-1.88	0.28	2.58E-08
SLC14A2	31.7	-1.88	0.42	0.000361
ASPN	71.2	-1.87	0.39	0.000102
LOC100524786	20.6	-1.87	0.48	0.002592
TFPI2	11.4	-1.86	0.57	0.017033
SHE	4.4	-1.86	0.54	0.011492
LOC100621260	12849.7	-1.86	0.36	2.46E-05
IFRD2	786.0	-1.86	0.32	2.01E-06
SNX15	163.0	-1.85	0.34	5.22E-06
TRIM15	8.0	-1.85	0.46	0.001776
SMOX	1788.2	-1.85	0.32	1.81E-06
SERINC2	456.0	-1.85	0.34	8.87E-06
DNM3	250.7	-1.84	0.35	1.56E-05
KLHL36	1087.4	-1.84	0.34	9.69E-06
MAP6	4.4	-1.84	0.59	0.027814
PSMF1	7549.4	-1.83	0.32	3.01E-06
LOC106510114	64.2	-1.83	0.37	5.13E-05
LOC110256393	3.4	-1.83	0.59	0.027959
LOC100627608	11.2	-1.82	0.41	0.00041
LOC102166347	1769.8	-1.82	0.32	3.11E-06
ICAM4	308.4	-1.81	0.28	9.62E-08
COL14A1	12.9	-1.81	0.50	0.00652
ARMC12	9.9	-1.81	0.44	0.001526
KIAA1257	1.6	-1.80	0.63	0.049722
INMT	1877.9	-1.80	0.42	0.000857
RNF10	42014.8	-1.79	0.33	7.35E-06
SMIM5	67.9	-1.79	0.37	8.98E-05
LOC106505356	18.2	-1.79	0.34	1.56E-05
RIOK3	13591.6	-1.79	0.30	7.56E-07
GABARAPL2	12153.5	-1.79	0.32	3.56E-06
LOC110261482	49.1	-1.78	0.34	1.76E-05
C8H4orf33	415.9	-1.78	0.30	1.11E-06
LOC100525237	34.1	-1.78	0.36	5.62E-05
SCUBE3	6.6	-1.78	0.56	0.022646
LOC100511509	441.2	-1.78	0.28	2.53E-07
LOC110255856	9.7	-1.78	0.46	0.003206
CD82	1126.8	-1.77	0.33	7.78E-06
ATG4A	2019.4	-1.77	0.33	7.71E-06
PLEK2	24.0	-1.77	0.36	6.24E-05
LOC102162202	89.1	-1.77	0.41	0.000607
TLX2	3.5	-1.77	0.62	0.049728
TARM1	79.1	-1.76	0.35	2.95E-05
CAPN11	42.0	-1.76	0.37	0.000102
CENPV	29.3	-1.76	0.47	0.004968
LOC100155734	2.7	-1.76	0.48	0.005915
LOC106509010	7.8	-1.75	0.54	0.020112
ADIPOR1	4557.5	-1.75	0.29	7.56E-07

PNPLA2	653.3	-1.75	0.36	0.000104
LOC110257355	8.5	-1.75	0.42	0.001284
COL4A1	10.0	-1.74	0.53	0.017049
CHIT1	355.0	-1.74	0.43	0.001824
RAB3IL1	277.8	-1.74	0.34	3.26E-05
LAMA3	2.6	-1.74	0.51	0.011909
MAD2L1BP	1080.3	-1.73	0.31	5.85E-06
LOC110261243	2.6	-1.72	0.56	0.029192
COL1A1	121.4	-1.72	0.58	0.038989
CHAC2	345.1	-1.72	0.30	2.65E-06
CRYBA4	266.1	-1.72	0.35	7.24E-05
HBM	14112.8	-1.71	0.35	7.24E-05
LOC106507598	8.8	-1.71	0.44	0.002821
UROD	726.1	-1.70	0.30	2.01E-06
SAMD7	10.3	-1.70	0.45	0.004595
RIMBP2	18.4	-1.69	0.41	0.001187
LOC102166303	14.9	-1.69	0.38	0.000467
MKRN1	9430.9	-1.69	0.30	2.80E-06
HBB	10582850.0	-1.69	0.33	2.46E-05
CCDC84	428.7	-1.69	0.28	6.76E-07
FAM178B	595.8	-1.68	0.46	0.006202
RETN	112.2	-1.68	0.36	0.000232
LOC102161780	2908.1	-1.67	0.36	0.000256
LOC100520032	33.3	-1.67	0.41	0.001596
LOC110258710	247.3	-1.67	0.46	0.006139
LOC110261569	18.1	-1.67	0.46	0.007054
S100A12	3210.9	-1.66	0.45	0.005804
WFDC3	62.0	-1.66	0.31	1.33E-05
ERFE	30.1	-1.65	0.40	0.001209
SS18L2	680.9	-1.65	0.31	1.00E-05
NTAN1	11002.5	-1.65	0.31	1.12E-05
LOC110261527	12.1	-1.65	0.32	1.81E-05
DYRK3	604.1	-1.65	0.33	5.48E-05
CLEC3B	7.5	-1.64	0.48	0.012581
BABAM1	3381.7	-1.64	0.29	2.75E-06
TNFAIP6	65.4	-1.64	0.31	1.33E-05
GPX3	1726.5	-1.63	0.50	0.017619
DGAT2	1464.6	-1.63	0.26	2.53E-07
DNAJC22	5.7	-1.63	0.53	0.030571
INO80C	528.4	-1.63	0.30	6.32E-06
LOC100515788	306322.2	-1.62	0.32	2.79E-05
GP2	22.1	-1.62	0.50	0.01964
FMOD	3.2	-1.62	0.53	0.032996
UPP1	1083.8	-1.61	0.39	0.001338
UBAC1	1643.6	-1.60	0.33	6.89E-05
INPP5J	3.1	-1.60	0.44	0.007302
LOC110260994	1143.1	-1.60	0.31	2.18E-05
S100A9	3874.4	-1.59	0.48	0.015201
LOC100158121	2.0	-1.59	0.53	0.036001

TAX1BP1	6785.7	-1.58	0.29	6.26E-06
FAM110B	18.9	-1.58	0.35	0.00041
ASNS	572.6	-1.57	0.34	0.000173
UBE2B	2789.0	-1.57	0.27	1.27E-06
EIF1	10879.0	-1.57	0.28	4.37E-06
LOC100525099	212.4	-1.56	0.36	0.000542
FBN3	3.3	-1.56	0.51	0.031441
MARCH2	7877.9	-1.56	0.28	5.13E-06
S100A8	2009.7	-1.56	0.46	0.011967
ATPIF1	36400.0	-1.56	0.27	2.01E-06
FAM214B	1660.4	-1.55	0.25	2.53E-07
RPS6KB2	874.7	-1.55	0.30	1.80E-05
RBX1	4365.9	-1.55	0.27	3.08E-06
KIAA1107	441.9	-1.54	0.35	0.000389
SMIM10	8.2	-1.54	0.30	2.46E-05
LOC102158889	23.7	-1.54	0.35	0.000467
LOC102164714	66.9	-1.53	0.53	0.04642
NARF	1675.2	-1.53	0.30	3.04E-05
TGM5	2.3	-1.53	0.49	0.026873
LOC106505550	64.5	-1.52	0.34	0.000302
LOC106507943	7.2	-1.52	0.38	0.001953
C16H5orf42	135.2	-1.52	0.34	0.000418
SLC7A10	10.0	-1.52	0.48	0.024854
LOC106505188	3.9	-1.52	0.47	0.020614
SEC62	3620.8	-1.52	0.28	1.06E-05
GSTK1	280.9	-1.52	0.44	0.012015
MAGI2	26.8	-1.51	0.41	0.006115
RNF123	2328.0	-1.51	0.31	9.74E-05
FBXO9	5015.4	-1.51	0.28	9.69E-06
LOC110261793	7.4	-1.51	0.48	0.023702
SPINK4	1996.6	-1.50	0.28	9.86E-06
CLIC2	344.6	-1.50	0.29	1.56E-05
SNX22	134.6	-1.50	0.36	0.001329
C17H20orf27	1980.6	-1.50	0.32	0.000173
NME4	480.2	-1.49	0.32	0.00015
LOC106508872	4.0	-1.48	0.51	0.046017
LOC110256043	436.8	-1.48	0.37	0.001688
LOC106504658	356.6	-1.48	0.37	0.001843
LOC110259135	124.7	-1.48	0.33	0.000298
FTH1	68230.4	-1.47	0.26	2.01E-06
UBE2H	12686.9	-1.47	0.23	2.02E-07
HBE1	1572.7	-1.46	0.44	0.014149
TRIM72	169.6	-1.46	0.29	4.89E-05
LOC100156381	16.4	-1.46	0.34	0.000835
YPEL5	4271.4	-1.46	0.24	4.46E-07
SIAH2	351.9	-1.46	0.25	1.41E-06
YPEL3	3588.6	-1.46	0.26	3.82E-06
LOC100524130	11.8	-1.46	0.49	0.035642
ARHGAP29	10.3	-1.46	0.41	0.007574

LOC110260749	155.7	-1.45	0.49	0.040255
LOC102168143	3.0	-1.45	0.39	0.004823
LOC110261115	23.3	-1.44	0.38	0.003966
SLC28A2	66.9	-1.44	0.39	0.005856
COX17	973.4	-1.44	0.29	5.96E-05
UQCR10	625.3	-1.44	0.27	9.69E-06
LOC110257408	168.5	-1.44	0.34	0.001163
CIR1	2319.4	-1.44	0.26	7.74E-06
KIAA1211L	8.9	-1.43	0.44	0.017649
LOC106509624	8.6	-1.43	0.36	0.002012
PBX1	153.7	-1.43	0.25	3.01E-06
MTMR11	73.2	-1.43	0.34	0.001102
PER1	489.3	-1.42	0.22	1.60E-07
LOC110256045	65.6	-1.42	0.38	0.004105
ENDOD1	1474.7	-1.42	0.27	1.83E-05
CCNJL	61.4	-1.42	0.28	4.87E-05
LOC110259958	351.8	-1.41	0.39	0.007773
NT5C3A	6507.0	-1.41	0.25	4.02E-06
FRMD4A	152.3	-1.40	0.27	1.56E-05
NTRK2	5.3	-1.40	0.43	0.018772
UROS	3568.3	-1.40	0.28	5.63E-05
RSPH9	274.3	-1.39	0.32	0.000552
CD14	1167.7	-1.38	0.32	0.000647
LOC100738720	4892.4	-1.38	0.30	0.000218
LOC102158679	43.4	-1.38	0.33	0.00122
ALOX5	780.9	-1.38	0.24	1.49E-06
LOC110261133	10.0	-1.37	0.34	0.001864
LOC100516852	135.4	-1.37	0.20	4.28E-08
LOC110261234	20.2	-1.37	0.41	0.013945
SHF	17.5	-1.36	0.34	0.002234
LOC102157461	24.6	-1.36	0.27	2.83E-05
FBXO7	1747.1	-1.36	0.25	1.11E-05
LOC110260737	34.4	-1.36	0.26	2.31E-05
ARHGEF26	2.5	-1.35	0.47	0.046672
PEF1	2658.0	-1.35	0.27	5.37E-05
CXCR6	34.4	-1.35	0.40	0.012125
MCOLN2	1116.0	-1.35	0.26	1.80E-05
CILP	4.3	-1.35	0.44	0.028317
PAGE2B	11.2	-1.35	0.46	0.041088
PTPRN	20.8	-1.35	0.35	0.003246
LOC106510004	2.7	-1.34	0.46	0.044529
FHDC1	35.7	-1.34	0.32	0.001099
PXDN	6.0	-1.33	0.43	0.028371
PLCL2	6450.3	-1.33	0.27	4.20E-05
SMAD9	17.4	-1.33	0.37	0.006528
PI16	14.1	-1.32	0.41	0.021802
MYOM2	158.2	-1.32	0.26	3.84E-05
ACKR4	6.0	-1.32	0.41	0.021764
LOC396866	19.6	-1.31	0.34	0.003124

CCDC28A	807.7	-1.31	0.29	0.000385
MAOB	134.7	-1.31	0.31	0.001149
CAV1	10.2	-1.30	0.33	0.002186
GSG1	12.3	-1.30	0.42	0.029749
PCSK9	9.0	-1.30	0.25	2.46E-05
RGS2	2267.5	-1.29	0.31	0.001209
COX7A1	28.9	-1.29	0.34	0.003797
GJA4	7.1	-1.29	0.37	0.011908
GYG1	3044.7	-1.28	0.23	3.82E-06
NFE2	3365.5	-1.28	0.25	3.09E-05
F7	21.5	-1.27	0.38	0.015089
LOC100524318	39.9	-1.27	0.32	0.002256
LOC106506455	54.8	-1.27	0.41	0.027864
H3F3A	17698.2	-1.27	0.25	2.46E-05
SOD3	31.1	-1.26	0.34	0.005142
LOC110257658	82.1	-1.26	0.27	0.000202
ANK1	334.9	-1.26	0.29	0.000563
PGLYRP1	63.5	-1.25	0.36	0.009992
EFNA1	4.6	-1.25	0.44	0.047886
GPX1	24658.8	-1.25	0.31	0.001789
ADAM19	5610.4	-1.25	0.21	7.56E-07
SLCO4C1	147.4	-1.25	0.33	0.003641
SGIP1	24.1	-1.25	0.27	0.000272
ITGB4	10.2	-1.24	0.38	0.016534
LOC100627410	13773.8	-1.24	0.23	1.00E-05
CCS	521.5	-1.24	0.25	3.69E-05
GABARAP	11431.1	-1.23	0.23	9.22E-06
GPAT4	2112.8	-1.23	0.20	7.03E-07
UBB	48079.5	-1.23	0.23	9.69E-06
LOC106510020	50.2	-1.23	0.32	0.002917
CITED4	16.1	-1.22	0.40	0.032864
ADAMTS2	36.4	-1.22	0.36	0.013258
SEPT12	131.6	-1.22	0.39	0.026193
PROK2	28.7	-1.22	0.42	0.045872
VPREB1	40.9	-1.21	0.35	0.0111
GPT	16.6	-1.21	0.37	0.016224
LOC102166222	2926.0	-1.21	0.22	8.10E-06
TRAK2	1707.9	-1.21	0.28	0.000486
GSTO1	342.5	-1.21	0.25	6.34E-05
GABARAPL1	5398.9	-1.21	0.22	7.28E-06
LOC106505536	497.1	-1.21	0.22	4.36E-06
LOC106509313	89.6	-1.21	0.28	0.000576
LOC110258635	7.3	-1.21	0.41	0.042532
MAFF	21.8	-1.20	0.37	0.018893
MICAL2	503.8	-1.20	0.29	0.001583
RBM38	1658.0	-1.20	0.31	0.00289
LOC100620627	112.8	-1.20	0.30	0.001843
SDSL	19.6	-1.20	0.37	0.020983
KPNA1	1411.0	-1.19	0.27	0.000439

CDKL1	20.1	-1.19	0.31	0.004047
HIST1H2BD	422.1	-1.19	0.29	0.001399
SMAP2	6231.2	-1.19	0.18	9.62E-08
MAP1LC3B	2192.9	-1.19	0.19	4.44E-07
ACKR1	38.1	-1.19	0.32	0.004801
ARL2BP	818.7	-1.19	0.19	4.52E-07
LOC110257651	1182.7	-1.19	0.27	0.000629
ALOX5AP	4748.5	-1.19	0.24	6.72E-05
MGP	29.4	-1.18	0.30	0.002448
RMND5A	3389.6	-1.18	0.28	0.000867
LOC100522725	1859.1	-1.18	0.27	0.000465
MMRN2	25.2	-1.18	0.25	0.000166
CRISPLD2	978.8	-1.18	0.19	2.07E-07
LOC110259710	76.5	-1.17	0.39	0.036293
NECTIN1	450.2	-1.17	0.23	2.19E-05
DNAJB2	1439.3	-1.17	0.21	4.26E-06
LOC110261477	20.7	-1.17	0.32	0.006726
DGKG	110.7	-1.17	0.23	2.12E-05
TSPAN33	41.3	-1.16	0.21	3.56E-06
E2F2	2998.1	-1.16	0.24	0.000126
ISG15	4560.2	-1.16	0.39	0.035462
LOC110258078	4.1	-1.16	0.38	0.032016
PTPA	1956.0	-1.16	0.23	5.62E-05
PPP2CB	1012.5	-1.15	0.25	0.000217
CAT	2193.1	-1.15	0.22	1.56E-05
ACSL6	87.7	-1.15	0.27	0.001116
CNN1	34.7	-1.15	0.33	0.011603
USP14	1676.1	-1.15	0.23	3.82E-05
CUL4A	2482.3	-1.15	0.26	0.00047
LOC100737631	272.8	-1.14	0.35	0.01735
NATD1	434.1	-1.14	0.32	0.008513
ATP5E	21480.0	-1.14	0.23	5.33E-05
LCN2	175.5	-1.14	0.35	0.018257
XPO7	3020.1	-1.13	0.28	0.001459
CTGF	14.7	-1.13	0.30	0.003799
FOXO3	2950.8	-1.13	0.23	5.08E-05
ROGDI	656.1	-1.13	0.29	0.002592
FBLN1	14.9	-1.13	0.37	0.033169
MYO1A	20.9	-1.13	0.28	0.00165
WASF3	15.2	-1.13	0.33	0.011893
NRP1	901.1	-1.12	0.23	8.85E-05
BEST3	55.2	-1.11	0.35	0.021022
LOC110257356	145.3	-1.11	0.26	0.000811
DUSP1	1447.3	-1.11	0.22	2.69E-05
LOC100737817	356.7	-1.11	0.20	7.71E-06
LOC100513767	8.7	-1.10	0.38	0.041718
GSDMB	11.3	-1.10	0.33	0.014164
KLF5	37.9	-1.10	0.31	0.008752
POU2F3	48.8	-1.10	0.37	0.036357

UBA52	46388.1	-1.10	0.21	1.33E-05
UBA52	46388.1	-1.10	0.21	1.33E-05
PRR13	2777.6	-1.10	0.28	0.002371
COL4A2	14.9	-1.10	0.38	0.049369
POLB	704.6	-1.09	0.25	0.000581
WBP2	3483.8	-1.09	0.21	3.04E-05
CCDC183	12.8	-1.09	0.35	0.025295
DCAF12	3423.0	-1.09	0.19	4.02E-06
RPS21	3951.7	-1.08	0.27	0.002457
HTR7	67.7	-1.08	0.25	0.000668
PPP1R3B	2175.0	-1.08	0.25	0.000848
GPNMB	176.4	-1.08	0.28	0.003173
SLC22A4	63.3	-1.07	0.27	0.002522
CEBPB	367.0	-1.07	0.33	0.018047
SERPINB1	8109.7	-1.07	0.23	0.000257
DYNLL1	2772.7	-1.07	0.23	0.000263
LOC100510923	882.5	-1.06	0.32	0.018023
C4H1orf162	727.9	-1.06	0.22	0.000151
KBTBD11	168.0	-1.06	0.21	3.69E-05
ENPP1	122.9	-1.05	0.28	0.003728
CNGA4	47.3	-1.05	0.36	0.045821
CLEC14A	16.8	-1.05	0.36	0.040128
LOC100158011	9558.5	-1.05	0.30	0.010966
NAT6	46.4	-1.05	0.33	0.021066
MLXIP	2881.7	-1.05	0.18	1.02E-06
PINK1	1319.8	-1.05	0.19	4.02E-06
HSD11B1	22.9	-1.05	0.29	0.00605
SLC38A5	280.4	-1.05	0.25	0.000815
LOC100625850	192.6	-1.04	0.23	0.000218
RGS16	13.7	-1.04	0.35	0.03629
FKBP5	5161.1	-1.04	0.21	8.06E-05
LOC102161418	344.3	-1.04	0.35	0.035421
MEDAG	50.1	-1.04	0.24	0.000688
TGM1	662.7	-1.04	0.30	0.011034
CHRNB4	13.4	-1.03	0.35	0.038669
PEAR1	15.0	-1.03	0.28	0.006128
VASH2	29.2	-1.03	0.26	0.002112
CD36	101.2	-1.03	0.32	0.020256
ACSL1	5656.9	-1.02	0.22	0.000273
HMG20B	591.2	-1.02	0.23	0.000368
CYP27A1	21.2	-1.02	0.31	0.019295
ABCB6	93.1	-1.02	0.23	0.000417
CRHBP	43.2	-1.02	0.24	0.000886
PSMD4	2689.2	-1.02	0.23	0.000356
IL18RAP	1411.3	-1.02	0.24	0.000753
MOB2	494.4	-1.01	0.23	0.000419
NT5DC2	284.8	-1.01	0.26	0.002575
ATP6V1E1	3619.4	-1.01	0.20	5.41E-05
DBN1	295.6	-1.01	0.21	7.06E-05

FILIP1	15.4	-1.01	0.33	0.031708
SLA	3063.4	-1.01	0.18	2.80E-06
SLC23A3	175.4	-1.01	0.18	6.02E-06
MCEMP1	1586.9	-1.01	0.31	0.021315
C4BPA	2203.8	-1.01	0.28	0.00605
NFKBIA	989.8	-1.01	0.18	4.21E-06
PAK6	6.4	-1.00	0.31	0.01964
NPRL3	205.0	-1.00	0.32	0.025665
TLCD2	188.0	-1.00	0.22	0.000418
RFESD	241.6	-1.00	0.21	9.90E-05
RHBDD1	744.6	-1.00	0.25	0.00253
OAZ1	23220.7	-0.99	0.21	0.000117
ARF4	4197.4	-0.99	0.23	0.00054
SLC19A1	76.6	-0.99	0.28	0.009992
GLRX5	616.8	-0.99	0.22	0.000468
ARHGEF37	72.0	-0.99	0.33	0.040033
PICALM	6294.7	-0.99	0.23	0.000651
LOC110256218	3187.6	-0.99	0.27	0.005879
LOC110257901	82.6	-0.98	0.21	0.000216
CYB5R1	312.1	-0.98	0.19	3.61E-05
EIF1B	1208.7	-0.98	0.24	0.001149
PLA2G7	36.4	-0.98	0.26	0.004698
LOC110257422	75.1	-0.98	0.29	0.011887
DLEC1	15.0	-0.98	0.24	0.001544
LOC102161750	3.9	-0.98	0.33	0.040033
ADGRG3	742.7	-0.98	0.27	0.007433
SELENOK	722.1	-0.98	0.20	8.30E-05
CNKSR3	138.5	-0.98	0.24	0.002107
PTPN3	34.5	-0.98	0.27	0.006741
REPS2	52.5	-0.98	0.17	3.44E-06
TMEM88	39.4	-0.97	0.23	0.000705
REC8	765.7	-0.97	0.22	0.000339
CAVIN1	22.1	-0.97	0.27	0.006742
LOC110255749	16.0	-0.97	0.32	0.035839
LOC110260261	129.8	-0.97	0.25	0.002565
ORMDL3	956.6	-0.97	0.21	0.000315
USF2	2093.3	-0.96	0.23	0.000833
NOCT	227.7	-0.96	0.23	0.001163
MYL9	8503.5	-0.96	0.25	0.003262
ACVR1B	172.0	-0.96	0.28	0.01122
PLPPR2	122.2	-0.95	0.28	0.012769
TPT1	464847.5	-0.95	0.21	0.000251
C10H9orf153	25.1	-0.95	0.27	0.009624
KIAA1522	47.3	-0.95	0.25	0.003262
CDHR3	79.9	-0.95	0.31	0.031322
HERPUD2	1536.5	-0.94	0.19	3.35E-05
C5AR2	165.9	-0.94	0.23	0.001776
ARG2	372.8	-0.94	0.27	0.008443
PTCRA	25.1	-0.94	0.30	0.025656

TINAGL1	1032.3	-0.94	0.21	0.000384
SEPT4	29.8	-0.94	0.26	0.006152
IFITM3	5871.7	-0.94	0.25	0.004575
SNX3	4369.7	-0.93	0.19	6.36E-05
PADI4	2937.3	-0.93	0.25	0.005162
AHSP	20659.6	-0.93	0.30	0.030962
KLF9	20.5	-0.92	0.27	0.010098
PADI3	104.5	-0.92	0.32	0.043132
MEGF9	2006.7	-0.92	0.29	0.02275
LOC110262257	25.4	-0.92	0.29	0.026621
CAPNS1	7683.9	-0.92	0.21	0.000758
TMEM40	245.0	-0.92	0.25	0.007283
GHITM	1180.5	-0.91	0.24	0.004273
CPT1A	2414.5	-0.91	0.22	0.00124
MYOM1	111.6	-0.91	0.29	0.024708
PPP1R15A	240.4	-0.91	0.21	0.000811
CAPN5	364.4	-0.91	0.25	0.006825
COX7A2	1226.6	-0.91	0.20	0.00034
SLC22A23	49.3	-0.91	0.31	0.040014
CWF19L2	1677.6	-0.91	0.21	0.000694
WNK4	188.4	-0.91	0.24	0.004539
AK2	2579.7	-0.90	0.19	0.000166
ATOH7	578.0	-0.90	0.25	0.006235
ANG	955.9	-0.90	0.21	0.000627
FOLR1	9138.4	-0.90	0.24	0.005061
RBPMS2	144.8	-0.90	0.27	0.017093
CMC4	45.4	-0.90	0.26	0.012951
PDZK1IP1	668.8	-0.90	0.27	0.015185
MGAM	884.5	-0.90	0.25	0.008957
TREM1	2559.5	-0.90	0.21	0.000841
LOC102158108	8.1	-0.89	0.29	0.028371
LOC100521322	110.4	-0.89	0.13	4.78E-08
LOC100158003	276.8	-0.89	0.27	0.016687
LOC110261673	63.5	-0.89	0.27	0.017619
UBL5	2018.7	-0.89	0.17	2.19E-05
CEBPD	1436.2	-0.89	0.21	0.000815
FCN1	3957.1	-0.89	0.25	0.009298
BNIP3L	12112.4	-0.89	0.19	0.000263
LOC110260336	39.4	-0.89	0.25	0.008743
SULF1	123.8	-0.89	0.27	0.017033
WIPI1	104.3	-0.89	0.22	0.002256
TLN2	16.8	-0.88	0.28	0.024025
LRG1	1203.2	-0.88	0.23	0.004311
DHRS4	531.2	-0.88	0.24	0.006115
DDAH1	1455.7	-0.88	0.24	0.006148
SAMSN1	3147.1	-0.88	0.22	0.002186
MAX	5667.0	-0.88	0.19	0.000263
FBXW4	612.2	-0.88	0.20	0.00057
LOC106510642	46.4	-0.87	0.26	0.016687

CSF2RA	1517.4	-0.87	0.21	0.001346
TUBA4A	4324.5	-0.87	0.19	0.000368
LOC110256379	7936.7	-0.87	0.24	0.00713
OSCAR	392.9	-0.87	0.24	0.00619
TNNI3	15.3	-0.87	0.27	0.021404
TSPAN2	206.6	-0.87	0.22	0.002864
CDH13	25.2	-0.87	0.25	0.011147
IL1RAP	1150.1	-0.87	0.27	0.020063
MINPP1	167.5	-0.87	0.23	0.003677
TAL1	187.4	-0.86	0.20	0.000467
PPM1M	1570.6	-0.86	0.15	2.86E-06
VNN3	745.9	-0.86	0.25	0.011954
PLIN5	60.1	-0.86	0.24	0.008922
EAF1	2710.3	-0.86	0.23	0.003766
PCYT1B	31.6	-0.86	0.24	0.008445
OTUB2	69.0	-0.86	0.30	0.047204
DRC1	421.5	-0.86	0.23	0.005127
RPLP1	20922.6	-0.86	0.22	0.003704
CD9	1363.2	-0.85	0.22	0.002697
MGLL	171.7	-0.85	0.22	0.003546
ZBTB8A	24.0	-0.85	0.23	0.004158
MAPK14	5924.9	-0.85	0.17	2.69E-05
SEPT5	390.3	-0.85	0.25	0.011219
ERG	98.9	-0.85	0.21	0.001291
IRS2	368.2	-0.85	0.17	7.24E-05
PARVB	1895.3	-0.85	0.21	0.002026
TLR4	1851.9	-0.85	0.22	0.003896
LOC100622710	1987.0	-0.85	0.21	0.002168
PHACTR3	17.4	-0.85	0.28	0.03428
TALDO1	2920.1	-0.85	0.25	0.014164
LOC110255267	154.0	-0.85	0.28	0.033169
MAGIX	20.4	-0.85	0.23	0.006862
RPLP0	57336.7	-0.84	0.19	0.000467
MYBPC2	8.0	-0.84	0.28	0.031708
MAPK13	486.9	-0.84	0.21	0.001897
FGFR1	35.9	-0.84	0.19	0.000576
LOC100517731	244.9	-0.84	0.23	0.006222
TPM1	1438.8	-0.84	0.21	0.002209
LZTS2	92.5	-0.84	0.26	0.022106
WDTC1	1839.5	-0.84	0.23	0.005974
ARHGEF40	52.7	-0.84	0.21	0.001734
ISCU	2262.6	-0.83	0.18	0.000185
C2H19orf35	39.4	-0.83	0.28	0.035103
LRRC4	14.7	-0.83	0.28	0.042293
GPR160	123.4	-0.83	0.24	0.009062
BABAM2	972.9	-0.83	0.19	0.00068
LOC102159995	11.9	-0.83	0.25	0.017093
HDC	50.4	-0.83	0.28	0.035355
LOC102164588	23.7	-0.83	0.25	0.017093

PET100	266.0	-0.83	0.19	0.000474
LMNA	173.7	-0.83	0.23	0.006825
SHB	65.7	-0.83	0.25	0.0137
UCP2	6992.0	-0.83	0.24	0.0111
GAPDH	19126.9	-0.83	0.19	0.000664
STOM	964.5	-0.83	0.14	2.01E-06
BTG3	94.6	-0.83	0.20	0.001064
C1RL	246.4	-0.83	0.25	0.014231
CDA	509.9	-0.83	0.23	0.006385
NCOA7	1352.0	-0.83	0.24	0.009992
MAST2	138.9	-0.83	0.26	0.023836
PPP2R3C	961.8	-0.83	0.18	0.000222
MED8	373.9	-0.82	0.21	0.002408
WNT5B	416.8	-0.82	0.20	0.00124
FAM160A2	529.0	-0.82	0.24	0.013707
C5AR1	4299.8	-0.82	0.18	0.000418
PLAUR	183.6	-0.82	0.25	0.015089
SLC22A7	148.1	-0.82	0.22	0.004698
SKP1	4286.7	-0.82	0.16	4.97E-05
MRVI1	154.3	-0.82	0.26	0.025797
MAP3K19	38.8	-0.81	0.20	0.00154
LOC110259329	3165.2	-0.81	0.21	0.003246
PITHD1	2161.9	-0.81	0.16	2.33E-05
AUH	520.2	-0.81	0.18	0.000337
FAM149A	407.0	-0.81	0.24	0.012951
LRRC51	30.9	-0.81	0.26	0.023836
RPS6KA2	277.1	-0.81	0.20	0.001288
LGALS8	2851.8	-0.81	0.18	0.000342
LOC110260298	1259.6	-0.81	0.25	0.020112
SVIP	11.3	-0.81	0.27	0.034428
GNAS	22341.3	-0.81	0.21	0.003246
SLC26A8	52.3	-0.81	0.24	0.014134
AP2M1	4244.3	-0.81	0.18	0.000337
TXNIP	17945.5	-0.81	0.23	0.008693
SLC25A37	734.0	-0.81	0.18	0.000265
RALB	2703.4	-0.81	0.15	1.81E-05
CST3	556.5	-0.80	0.27	0.036001
EIF3K	6647.4	-0.80	0.17	0.000102
SRXN1	189.3	-0.80	0.19	0.000758
LOC102160213	26.0	-0.80	0.26	0.030388
JDP2	217.9	-0.80	0.19	0.000901
EFCAB2	29.7	-0.80	0.24	0.017734
ASAP3	2970.8	-0.80	0.21	0.004767
ZMAT5	111.6	-0.79	0.23	0.011893
IL3RA	68.6	-0.79	0.26	0.029749
LOC102161210	17.9	-0.79	0.20	0.002256
CREG1	1875.6	-0.79	0.21	0.003688
SULT1A3	1353.8	-0.79	0.23	0.01159
LOC102168178	144.1	-0.79	0.27	0.040183

FKBP8	3941.7	-0.79	0.21	0.004047
GNAZ	203.2	-0.79	0.26	0.034795
LOC110256540	33.8	-0.79	0.20	0.002477
LOC100519082	4002.3	-0.79	0.27	0.045935
ITGA2B	2128.8	-0.78	0.25	0.02367
LOC100519934	3559.1	-0.78	0.24	0.021022
KIAA1191	3233.1	-0.78	0.15	1.56E-05
MXD1	14283.9	-0.78	0.20	0.002702
WDR45	762.6	-0.78	0.17	0.000272
ATP7B	16.5	-0.78	0.27	0.041612
BOLA3	360.5	-0.77	0.19	0.001394
PIM1	6817.9	-0.77	0.17	0.000289
MXRA7	284.6	-0.77	0.25	0.027958
ATP9A	220.7	-0.77	0.21	0.004698
TMEM59	4015.9	-0.77	0.15	3.79E-05
VPS37C	238.2	-0.77	0.21	0.00706
GPR84	2269.0	-0.77	0.27	0.049263
BST1	74.1	-0.77	0.26	0.035421
LOC102157641	37.2	-0.77	0.23	0.017093
CAPN3	1524.7	-0.77	0.23	0.014136
TP53INP1	5493.3	-0.77	0.17	0.000339
LOC110261656	35.9	-0.77	0.26	0.037647
LOC100736623	1388.6	-0.76	0.21	0.008003
MAP2K3	1361.3	-0.76	0.21	0.005879
EIF2AK1	1392.7	-0.76	0.20	0.003703
OGFRL1	1527.3	-0.76	0.17	0.00055
MMP25	1053.2	-0.76	0.23	0.016091
WWC2	64.8	-0.76	0.24	0.022175
UBE2L6	1302.4	-0.76	0.26	0.043644
MAP1LC3A	190.0	-0.75	0.20	0.004364
TMOD1	3238.8	-0.75	0.18	0.000815
LOC110260061	43.7	-0.75	0.22	0.010578
BMX	478.5	-0.75	0.19	0.002311
CERS4	1992.6	-0.75	0.19	0.003174
NUP50	2672.9	-0.75	0.17	0.000463
LOC110256683	311.7	-0.75	0.20	0.004363
CIART	21.0	-0.75	0.22	0.013387
CAVIN2	134.6	-0.75	0.25	0.03478
CDKN2D	839.6	-0.75	0.18	0.001729
PQBP1	466.5	-0.75	0.20	0.004981
THBS1	9640.1	-0.75	0.25	0.04026
TSPYL1	1233.4	-0.75	0.21	0.008503
NFIL3	1934.8	-0.74	0.19	0.002026
SET	11646.1	-0.74	0.20	0.005951
LOC106509194	26.4	-0.74	0.25	0.035421
LOC106505679	55.6	-0.74	0.24	0.025547
UBL7	564.7	-0.74	0.18	0.00156
NFAM1	484.3	-0.74	0.22	0.014894
RNF14	1116.9	-0.74	0.16	0.00023

TUBB1	4003.1	-0.74	0.21	0.011506
VDR	453.8	-0.74	0.21	0.008255
IFIT1	23706.4	-0.74	0.25	0.035421
TKT	2726.7	-0.74	0.19	0.004056
KIAA1147	2632.6	-0.74	0.22	0.014102
C4H1orf226	48.5	-0.74	0.25	0.044366
PFKFB2	1088.6	-0.73	0.21	0.00803
TBX6	136.0	-0.73	0.24	0.032701
FAM135A	736.0	-0.73	0.25	0.036357
MUC19	1302.4	-0.73	0.24	0.02809
STAB1	367.0	-0.73	0.19	0.003248
SH3BGRL3	9351.1	-0.73	0.18	0.001199
LGALS9	2816.2	-0.73	0.25	0.044377
SAG	16.1	-0.73	0.23	0.020729
RHOC	1033.9	-0.73	0.18	0.001906
TLR9	166.4	-0.73	0.18	0.001432
MYBPH	1853.1	-0.73	0.22	0.014869
FZR1	1125.8	-0.72	0.21	0.009073
CDKN3	107.3	-0.72	0.21	0.013878
CSF3R	18813.2	-0.72	0.23	0.027959
HIPK1	7055.0	-0.72	0.19	0.003119
CCM2L	529.5	-0.72	0.22	0.015185
UBE2F	309.4	-0.72	0.21	0.011236
UBALD2	698.9	-0.72	0.22	0.016219
IL1B	314.0	-0.72	0.22	0.018814
RPLP2	6345.9	-0.72	0.22	0.016632
IFT20	198.4	-0.72	0.18	0.002822
SIGLEC5	4360.3	-0.71	0.17	0.000658
TTC7B	333.0	-0.71	0.20	0.007353
ONECUT1	13.9	-0.71	0.23	0.026177
CDKN2C	387.0	-0.71	0.19	0.004472
SOCS1	140.4	-0.71	0.22	0.02124
GNG11	1422.3	-0.71	0.22	0.017708
CCRL2	2339.1	-0.71	0.18	0.003677
SLC3A2	407.2	-0.71	0.20	0.007302
C7H6orf222	385.6	-0.71	0.20	0.008445
LOC100627949	740.0	-0.70	0.18	0.003021
TLR2	1142.3	-0.70	0.16	0.000418
CARHSP1	1116.0	-0.70	0.19	0.00526
AGER	42.1	-0.70	0.24	0.043552
DDIT4	1074.1	-0.70	0.17	0.001906
CDS1	144.7	-0.70	0.21	0.012401
KIAA0513	1417.3	-0.70	0.20	0.010966
LOC102164379	50.4	-0.70	0.24	0.039754
HGF	168.5	-0.70	0.21	0.015134
AP2B1	2489.7	-0.70	0.19	0.006742
AP3M2	150.5	-0.70	0.19	0.004442
HMOX1	557.3	-0.70	0.19	0.007818
STOML2	873.2	-0.69	0.18	0.004311

ARHGAP10	582.8	-0.69	0.20	0.012527
SYTL4	113.0	-0.69	0.23	0.035103
MARCH8	1442.1	-0.69	0.14	8.30E-05
HBS1L	1150.0	-0.69	0.19	0.007647
FBXO3	455.0	-0.69	0.20	0.010479
UBE2R2	2429.0	-0.69	0.17	0.002168
LOC100621455	1072.9	-0.69	0.17	0.001162
SLC17A8	12.8	-0.69	0.23	0.033169
PTGES3L	471.1	-0.69	0.19	0.005804
RPS11	36530.4	-0.69	0.15	0.000249
SMTN	117.2	-0.69	0.21	0.020488
RPS17	19751.8	-0.69	0.17	0.001163
ECM1	5864.4	-0.69	0.15	0.000361
LOC110255963	121.1	-0.68	0.22	0.031166
SERF2	6998.1	-0.68	0.17	0.002142
SLC40A1	4265.2	-0.68	0.15	0.000423
IFITM1	10340.4	-0.68	0.22	0.032404
FFAR2	1274.0	-0.68	0.22	0.030154
SIGLEC14	2783.8	-0.68	0.18	0.003815
MEIOB	115.1	-0.68	0.23	0.043722
PLA2G15	148.6	-0.67	0.24	0.047886
ECE1	1092.0	-0.67	0.19	0.007647
LOC106510586	26.3	-0.67	0.23	0.036078
MOB1B	781.9	-0.67	0.16	0.001555
ELOC	688.5	-0.67	0.16	0.000807
COL27A1	13.1	-0.67	0.22	0.032203
TP53INP2	133.4	-0.67	0.15	0.000576
ACTR10	1124.7	-0.67	0.20	0.012095
ABCB9	146.7	-0.67	0.19	0.009672
FARS2	61.5	-0.67	0.22	0.028893
PMF1	107.8	-0.67	0.18	0.005974
LRRC75A	137.8	-0.67	0.21	0.020557
ETFRF1	121.2	-0.66	0.20	0.02003
GAPT	443.7	-0.66	0.22	0.039402
UBXN6	370.1	-0.66	0.18	0.007456
PKP4	22.5	-0.66	0.19	0.010266
RPS24	49253.3	-0.66	0.19	0.007799
PHKA2	818.4	-0.66	0.17	0.002186
PDLIM1	2359.7	-0.66	0.22	0.035636
CDKN1A	412.7	-0.66	0.17	0.003242
PLIN3	404.9	-0.66	0.22	0.040796
RPL18A	9030.1	-0.66	0.19	0.009679
SLC11A1	715.8	-0.66	0.22	0.036268
ITGAM	2939.4	-0.66	0.16	0.002176
RPS2	22259.8	-0.65	0.20	0.015838
FAH	245.4	-0.65	0.21	0.033169
SLC26A6	871.3	-0.65	0.13	7.06E-05
COX6A1	1972.1	-0.65	0.14	0.000268
SLC1A5	737.8	-0.65	0.17	0.002864

YBX3	3926.1	-0.65	0.13	8.17E-05
ST13	4780.4	-0.64	0.15	0.000781
SGMS1	1275.4	-0.64	0.19	0.011219
NUDT7	178.0	-0.64	0.20	0.021404
DAGLB	666.7	-0.64	0.16	0.001548
UBE2C	304.6	-0.64	0.20	0.023486
IK	2366.3	-0.64	0.18	0.010078
E2F4	1278.9	-0.64	0.16	0.002627
SLC27A4	251.0	-0.64	0.21	0.03428
SARS	1724.3	-0.64	0.18	0.011379
F13A1	16079.0	-0.63	0.17	0.005715
SH3BP5	1895.1	-0.63	0.16	0.001772
PRAM1	2063.2	-0.63	0.19	0.014451
PLEKHB2	3961.7	-0.63	0.16	0.002189
FAM189A2	94.0	-0.63	0.20	0.021022
BOLA2B	347.4	-0.63	0.16	0.003743
SDS	19656.9	-0.63	0.22	0.047356
MAP3K9	638.9	-0.63	0.17	0.005951
LOC110261034	1339.8	-0.63	0.20	0.025417
PDLIM7	3045.5	-0.63	0.17	0.006864
CD44	7572.7	-0.63	0.12	3.18E-05
XCR1	605.1	-0.63	0.20	0.027959
BSDC1	1842.4	-0.63	0.19	0.017033
POLR2K	302.1	-0.63	0.17	0.005127
SLC2A3	11079.8	-0.63	0.21	0.032424
CCNI	6059.1	-0.62	0.15	0.001659
SLC7A5	515.2	-0.62	0.22	0.046483
LOC100518417	4173.0	-0.62	0.20	0.023172
DCUN1D1	1208.5	-0.62	0.16	0.003452
THBD	250.4	-0.62	0.21	0.043482
TNFSF13B	1487.2	-0.62	0.16	0.00219
LOC110256941	2216.1	-0.62	0.21	0.04515
PAIP2	3023.0	-0.62	0.16	0.0021
OST4	2391.6	-0.62	0.15	0.001864
SLC25A38	823.8	-0.62	0.15	0.001245
RUSC2	135.3	-0.62	0.19	0.020604
TXNL4B	1312.0	-0.62	0.16	0.003203
FOSL2	2575.3	-0.62	0.16	0.002267
CXCR2	10395.0	-0.62	0.20	0.033838
CGRRF1	279.7	-0.62	0.16	0.003174
TIMP3	999.8	-0.62	0.18	0.01531
EIF3F	2954.6	-0.62	0.18	0.011305
LOC110256794	210.2	-0.61	0.20	0.034688
CHI3L1	5108.5	-0.61	0.14	0.000815
IPMK	1880.4	-0.61	0.18	0.012103
RAB3D	2450.4	-0.61	0.17	0.005974
PGD	3762.4	-0.60	0.16	0.005083
LOC100622306	1663.1	-0.60	0.19	0.025207
ABTB1	534.7	-0.60	0.18	0.01735

CCR1	5893.2	-0.60	0.18	0.017535
BASP1	1572.9	-0.60	0.14	0.000692
CNR1	78.5	-0.60	0.18	0.013048
USP32	3281.9	-0.60	0.18	0.014231
AKR1B1	514.1	-0.60	0.15	0.001776
GADD45A	997.8	-0.60	0.20	0.038669
NUP155	848.2	-0.60	0.16	0.006519
GP6	945.4	-0.60	0.20	0.037877
PILRA	3142.0	-0.60	0.20	0.041298
PLOD1	467.1	-0.59	0.17	0.007302
RHOU	172.7	-0.59	0.20	0.039656
NHSL2	344.0	-0.59	0.19	0.023215
TTC1	673.3	-0.59	0.20	0.038285
GLRA3	46.2	-0.59	0.19	0.030766
CNPPD1	728.0	-0.59	0.18	0.014686
RAB8A	1797.7	-0.59	0.15	0.002883
ARAP3	1735.6	-0.59	0.18	0.01964
PXN	5426.3	-0.59	0.16	0.007108
LOC100738859	2378.1	-0.59	0.20	0.038989
ZER1	519.1	-0.58	0.19	0.029422
LOC106507710	67.9	-0.58	0.18	0.015539
CFDP1	1412.8	-0.58	0.17	0.011204
FAM204A	1310.0	-0.58	0.14	0.001555
STX11	1087.2	-0.58	0.19	0.031756
ALDOA	10513.1	-0.58	0.18	0.01964
RNASE4	631.8	-0.58	0.15	0.004364
AMPD2	1286.2	-0.58	0.18	0.020322
NOP10	1678.2	-0.58	0.13	0.000467
SH2D1B	145.6	-0.58	0.19	0.036215
SDE2	1482.0	-0.58	0.14	0.000835
BCL6	1779.6	-0.58	0.16	0.007538
CUX1	1516.6	-0.58	0.14	0.001695
MAPKAPK2	2625.6	-0.58	0.14	0.001078
ZC3H10	16239.1	-0.58	0.13	0.000371
ITGAX	6689.1	-0.57	0.15	0.004356
FBXO48	617.6	-0.57	0.17	0.012562
ALDH2	1242.5	-0.57	0.16	0.006818
VSIR	6510.0	-0.57	0.16	0.00978
FHL1	3102.9	-0.57	0.20	0.045547
ATP6V0A1	524.6	-0.57	0.17	0.013878
SH3BGRL2	2107.9	-0.57	0.17	0.017382
ARV1	402.8	-0.57	0.14	0.002448
RPS7	12050.2	-0.57	0.15	0.003797
ATP5J2	1056.8	-0.57	0.12	0.000101
LOC100515345	7381.5	-0.57	0.17	0.016943
ITGB5	555.5	-0.57	0.16	0.006537
ZFAND3	1202.7	-0.57	0.11	4.72E-05
RPS10	9187.3	-0.56	0.14	0.002235
CISD1	519.4	-0.56	0.14	0.001567

DNAJB6	1021.4	-0.56	0.13	0.000644
RAB3IP	382.6	-0.56	0.18	0.027626
IGF2BP2	397.2	-0.56	0.16	0.011219
LITAF	5586.4	-0.56	0.16	0.008364
LPIN2	3675.4	-0.56	0.14	0.001697
PNPLA8	744.2	-0.56	0.13	0.001053
LOC106504436	2321.8	-0.55	0.16	0.007832
FUNDC2	543.4	-0.55	0.14	0.002276
OGDH	2244.7	-0.55	0.19	0.043867
SSH2	15158.8	-0.55	0.16	0.011986
TGFA	2371.3	-0.55	0.17	0.023896
FOXO4	1340.2	-0.55	0.13	0.0005
LOC100517285	3191.9	-0.55	0.18	0.031742
FCER1G	5874.2	-0.55	0.18	0.030373
CCND3	5872.7	-0.55	0.14	0.002861
RPL24	9745.1	-0.55	0.14	0.003677
FAM210B	579.2	-0.55	0.15	0.004678
RPL12	12665.2	-0.55	0.14	0.002893
TGM2	576.2	-0.55	0.18	0.026625
WDR26	3575.5	-0.55	0.15	0.004798
NCK2	2154.8	-0.54	0.18	0.035043
COX5B	1086.9	-0.54	0.14	0.002633
CMC2	160.4	-0.54	0.14	0.002479
TMBIM6	13525.0	-0.54	0.12	0.000459
ZYX	8766.9	-0.54	0.17	0.017619
PCMT1	681.7	-0.54	0.13	0.001608
TNIP1	2147.5	-0.54	0.17	0.027021
COTL1	7294.3	-0.54	0.17	0.021572
RPL35	11267.1	-0.54	0.15	0.005309
LOC102158334	67.1	-0.54	0.16	0.011471
G6PC3	477.7	-0.54	0.18	0.04026
TMEM120A	530.5	-0.54	0.18	0.034688
EIF5	5864.0	-0.54	0.15	0.007647
AP2A2	1413.3	-0.54	0.19	0.0499
PROS1	513.3	-0.54	0.18	0.036293
ASPRV1	346.4	-0.53	0.13	0.002234
APOBR	4767.4	-0.53	0.14	0.004261
BBIP1	198.7	-0.53	0.16	0.016627
TBC1D8	123.3	-0.53	0.15	0.006459
RPL10	29250.2	-0.53	0.15	0.008713
PAM	1003.6	-0.53	0.17	0.030434
RAPSN	48.8	-0.53	0.18	0.036388
BCAS3	453.0	-0.53	0.16	0.016956
LIMK2	757.0	-0.53	0.11	0.000218
PFKFB4	713.0	-0.52	0.13	0.001745
SLC13A5	23.5	-0.52	0.17	0.031707
IL4R	3576.0	-0.52	0.15	0.008455
ACSL4	10247.3	-0.52	0.16	0.025922
LOC106509609	75.1	-0.51	0.18	0.043711

APEH	544.3	-0.51	0.14	0.005497
RPL36	3871.7	-0.51	0.16	0.025547
GMPPA	452.3	-0.51	0.16	0.020664
PSME4	2150.6	-0.51	0.13	0.002917
TWF2	1328.7	-0.51	0.16	0.019108
MBOAT2	157.6	-0.51	0.14	0.005011
ADAMTSL4	1150.9	-0.51	0.15	0.010078
KCNJ14	20.5	-0.51	0.16	0.022447
TMEM57	606.1	-0.51	0.18	0.047001
TPM4	14713.6	-0.51	0.17	0.03996
ERRFI1	48.0	-0.51	0.16	0.022199
PPOX	297.5	-0.51	0.13	0.002592
IVNS1ABP	7446.1	-0.50	0.15	0.015415
FRG1	607.3	-0.50	0.13	0.003568
SLC44A2	6905.9	-0.50	0.15	0.012103
LOC110255544	216.0	-0.50	0.12	0.001138
EHD3	719.5	-0.50	0.17	0.038285
NAPA	1513.5	-0.50	0.17	0.045935
PNPLA6	2709.2	-0.50	0.17	0.042385
RILPL2	2066.1	-0.49	0.14	0.01034
NDUFB8	782.5	-0.49	0.11	0.00041
PTP4A2	4647.5	-0.49	0.16	0.027814
PFKFB3	525.0	-0.49	0.14	0.012055
HSBP1	879.2	-0.49	0.13	0.005099
YIPF3	952.4	-0.49	0.14	0.007369
EXOC7	581.1	-0.48	0.16	0.040647
GAB2	1401.8	-0.48	0.16	0.03996
HCK	2733.3	-0.48	0.17	0.043711
RPL38	8044.0	-0.48	0.13	0.006032
DDB1	3053.9	-0.48	0.14	0.016534
INPP5A	477.6	-0.47	0.13	0.004712
LOC100525590	103.6	-0.47	0.16	0.033753
TTC13	1033.7	-0.47	0.13	0.005989
TCEANC	369.7	-0.47	0.14	0.013062
RPS20	18399.0	-0.47	0.15	0.032203
ETS2	1867.5	-0.47	0.16	0.04348
SORT1	1158.8	-0.47	0.15	0.027881
CREM	662.9	-0.47	0.13	0.008445
TSC22D3	15621.2	-0.47	0.13	0.009242
MBOAT1	2037.7	-0.47	0.13	0.006466
PPP1R3D	905.8	-0.47	0.13	0.010676
E2F3	535.1	-0.46	0.15	0.025852
RAB5C	1392.2	-0.46	0.13	0.005885
ZMAT2	1700.2	-0.46	0.13	0.009198
LOC102161631	60.3	-0.46	0.12	0.005162
ZFAND2B	494.0	-0.46	0.13	0.012117
GRIPAP1	1321.9	-0.46	0.13	0.007513
MYL6	8342.1	-0.46	0.16	0.039766
REPS1	941.0	-0.46	0.15	0.033081

ODC1	2653.9	-0.46	0.10	0.000344
ENSA	1185.3	-0.46	0.12	0.003338
BEND7	1113.9	-0.46	0.13	0.006818
ZRANB1	682.4	-0.45	0.13	0.011893
ITPKC	197.2	-0.45	0.12	0.00289
RPL31	10788.6	-0.45	0.14	0.01964
DAZAP2	10533.3	-0.45	0.10	0.000263
MPC2	447.0	-0.45	0.13	0.00841
MRPL22	320.9	-0.45	0.15	0.034428
IQSEC1	2520.9	-0.45	0.15	0.035103
STXBP2	3455.8	-0.45	0.14	0.027429
RETREG2	1140.7	-0.45	0.14	0.02582
RHOB	920.8	-0.45	0.15	0.033822
UBXN8	906.9	-0.44	0.13	0.012638
CORO1C	2077.1	-0.44	0.10	0.000263
SDHB	795.4	-0.44	0.12	0.005989
FHOD1	782.6	-0.44	0.13	0.011908
NSMCE1	378.4	-0.44	0.15	0.047242
LOC102165318	41.2	-0.44	0.15	0.042554
CSRNP1	561.4	-0.44	0.15	0.045436
LAPTM4A	2172.2	-0.44	0.14	0.025547
STAMBPL1	216.1	-0.44	0.11	0.002627
PINX1	165.4	-0.44	0.14	0.031826
LOC102166710	27.9	-0.44	0.15	0.044042
ARHGAP25	4890.8	-0.44	0.09	0.000229
PRPF18	812.5	-0.44	0.13	0.017093
CMIP	1109.8	-0.44	0.12	0.008693
LOC102161969	63.9	-0.43	0.13	0.010857
XPO6	7821.2	-0.43	0.14	0.035043
PLEK	7282.2	-0.43	0.13	0.014149
VASP	8365.9	-0.43	0.14	0.028649
IFRD1	915.7	-0.43	0.15	0.039808
PSMC3	1417.4	-0.43	0.14	0.031356
IRF9	1814.9	-0.43	0.15	0.045436
EIF3H	8216.0	-0.43	0.13	0.014382
EIF3G	3105.1	-0.42	0.14	0.042554
UBE2D3	2033.4	-0.42	0.13	0.015374
VPS26B	1469.5	-0.42	0.11	0.005324
MYD88	978.0	-0.42	0.13	0.026942
STK40	1549.1	-0.42	0.13	0.022392
SIRPA	2823.0	-0.42	0.14	0.042988
SHISA5	3811.4	-0.41	0.14	0.034795
SCNM1	329.9	-0.41	0.14	0.043032
AHCY	990.9	-0.41	0.10	0.001596
SCRG1	272.4	-0.41	0.14	0.039561
MRPS18A	445.6	-0.41	0.14	0.048764
HIP1R	1858.2	-0.41	0.11	0.00711
RPS26	8961.4	-0.41	0.12	0.018831
PSORS1C2	50.4	-0.41	0.14	0.048219

LOC100524873	1306.0	-0.41	0.12	0.014803
NUDT4	955.4	-0.41	0.09	0.000128
EGLN3	834.6	-0.40	0.10	0.002382
TCP11L2	2716.5	-0.40	0.13	0.035103
C1H9orf40	207.2	-0.40	0.13	0.026625
SEMA5A	75.9	-0.40	0.12	0.009992
LDHA	7124.3	-0.40	0.13	0.036799
RPL19	17117.6	-0.40	0.13	0.035043
PDLIM5	317.0	-0.40	0.14	0.049693
NT5C3B	232.0	-0.40	0.13	0.026625
RPS12	19449.1	-0.40	0.13	0.027266
RPL37	14962.7	-0.40	0.12	0.014797
LOC110255801	48.2	-0.39	0.13	0.031888
TSG101	1286.5	-0.39	0.12	0.026625
PTPN1	4892.5	-0.39	0.10	0.001776
IRF2BPL	770.9	-0.38	0.12	0.02733
ATP5L	1251.5	-0.38	0.12	0.017093
IFNGR2	2104.4	-0.38	0.13	0.036001
HACD4	1084.4	-0.38	0.12	0.023214
RPL23A	26966.7	-0.38	0.12	0.022447
CALCOCO1	1424.9	-0.38	0.11	0.014566
ADGRE5	13433.4	-0.38	0.12	0.027814
TNFRSF1B	8137.8	-0.37	0.13	0.044813
IKBKB	1575.7	-0.37	0.11	0.015941
TIMM10	207.2	-0.37	0.13	0.042193
C8H4orf32	340.2	-0.37	0.12	0.027959
SLC25A51	3971.2	-0.37	0.12	0.035043
FGD3	5768.5	-0.37	0.12	0.031873
CRTC2	1105.7	-0.36	0.12	0.03646
UBC	16587.9	-0.36	0.12	0.032016
LOC110260250	98.5	-0.36	0.09	0.002477
PRKAB1	838.7	-0.36	0.12	0.044446
GANC	288.2	-0.36	0.12	0.040873
GDI1	3805.7	-0.36	0.12	0.042049
SCARB1	532.7	-0.36	0.12	0.040774
ASNA1	462.0	-0.36	0.11	0.027321
COPG1	2738.1	-0.35	0.12	0.049911
PPIL2	1241.5	-0.35	0.09	0.004004
DCAF8	2143.0	-0.35	0.11	0.026439
CHMP4B	1169.8	-0.35	0.11	0.030451
OTUD5	2114.1	-0.35	0.11	0.018893
FAF1	426.4	-0.35	0.11	0.033397
OLFML3	267.7	-0.34	0.10	0.015539
RPL11	16259.9	-0.34	0.11	0.030479
LRRC28	487.2	-0.34	0.11	0.028095
ACAA1	1042.4	-0.34	0.11	0.026623
PIK3IP1	1356.0	-0.34	0.11	0.032181
LSM12	518.0	-0.33	0.10	0.010007
BTF3	5704.7	-0.33	0.11	0.03904

LOC733637	11163.9	-0.33	0.10	0.023371
ATP5J	1042.7	-0.33	0.11	0.038039
LOC110260596	128.5	-0.32	0.10	0.015831
GLYR1	2601.2	-0.32	0.11	0.039451
NDEL1	1216.1	-0.32	0.11	0.034162
ATF4	4169.8	-0.31	0.09	0.005715
LMBR1L	1384.7	-0.31	0.11	0.046106
MRPS25	627.3	-0.31	0.10	0.031352
FAM32A	1470.9	-0.31	0.10	0.031707
MSL3	1375.5	-0.31	0.10	0.033251
NDUFB4	716.8	-0.30	0.10	0.048219
RFFL	2876.7	-0.30	0.08	0.00566
ANXA11	2895.2	-0.29	0.10	0.04908
SYK	2678.4	-0.29	0.08	0.007456
DEGS1	593.9	-0.29	0.09	0.030373
APMAP	1408.1	-0.29	0.09	0.018122
MAPRE2	2529.1	-0.29	0.09	0.023896
ACTN1	3666.5	-0.28	0.09	0.032016
MFN2	727.6	-0.28	0.09	0.032996
ANKS1A	354.3	-0.28	0.09	0.035421
RAB9A	470.5	-0.27	0.09	0.027959
IP6K1	2159.4	-0.27	0.08	0.01964
FAM155B	1240.0	-0.25	0.09	0.047025
FGFR1OP	270.8	-0.25	0.08	0.036804
TAF8	755.3	-0.24	0.07	0.009486
STK24	3415.6	-0.24	0.07	0.017033
RALBP1	1531.3	-0.23	0.07	0.027959
CRIPT	735.0	-0.22	0.07	0.034059
WBP1L	1425.1	-0.22	0.06	0.003943
CCDC69	5237.1	-0.22	0.06	0.011909
Post-weaning				
SUCNR1	25.8	2.12	0.48	0.000439
LOC100154128	1.7	2.00	0.69	0.04341
SBSPON	10.6	1.84	0.41	0.000351
SLAMF8	34.5	1.48	0.52	0.047051
LOC100522787	37.0	1.40	0.34	0.00121
LOC102161330	31.7	1.35	0.27	4.63E-05
MET	6.5	1.34	0.39	0.011745
TIGIT	248.9	1.32	0.35	0.004472
KITLG	3.7	1.20	0.38	0.024952
NEFH	13.7	1.19	0.38	0.028956
SLCO2B1	62.1	1.17	0.32	0.005631
PADI2	146.5	1.07	0.29	0.005885
LOC100737823	8.9	1.06	0.37	0.044112
PYGO1	14.3	1.05	0.31	0.011528
PSAT1	21.4	1.03	0.34	0.033753
TDRKH	9.9	0.98	0.29	0.014217
LOC106505804	67.7	0.98	0.22	0.000361
SYT11	67.7	0.97	0.32	0.031322

NPR3	168.9	0.93	0.30	0.030373
CCDC63	21.1	0.89	0.26	0.012519
LOC102165774	144.3	0.88	0.29	0.031899
RRAGD	43.3	0.84	0.24	0.008036
CASC1	124.1	0.82	0.23	0.009665
MAPK4	40.9	0.80	0.27	0.037998
PTPRO	203.1	0.80	0.18	0.000647
LOC110255290	168.7	0.80	0.21	0.004039
PTPN4	944.4	0.76	0.24	0.021443
MITF	165.0	0.74	0.16	0.000266
DAPK2	241.1	0.74	0.23	0.023009
PFN2	39.0	0.70	0.20	0.007302
LOC100620407	219.2	0.70	0.19	0.005885
LOC102162428	15.3	0.69	0.23	0.037232
LOC102165892	18.1	0.66	0.18	0.006666
LYSMD2	36.9	0.64	0.13	4.63E-05
DUSP14	115.6	0.64	0.20	0.018321
DLG3	91.4	0.63	0.18	0.011573
SQLE	233.9	0.62	0.15	0.001083
LOC106504408	17.2	0.60	0.18	0.013707
ASPH	108.0	0.57	0.17	0.013258
LOC100522818	29.6	0.57	0.17	0.015968
CD200R1	209.5	0.55	0.18	0.026549
F2R	453.3	0.55	0.19	0.042554
LOC102163364	32.2	0.55	0.18	0.033834
GPR15	242.5	0.54	0.19	0.04879
LOC110257567	19.3	0.54	0.16	0.01375
LOC100626606	95.1	0.53	0.16	0.019743
FANCL	46.6	0.53	0.13	0.002168
KIAA1328	25.1	0.52	0.16	0.017093
RASGRP3	386.4	0.52	0.15	0.010334
KMO	247.3	0.51	0.13	0.003447
BHLHE40	728.6	0.51	0.16	0.029201
SCD	296.5	0.50	0.16	0.027429
SEPT3	748.6	0.50	0.11	0.000481
SC5D	220.2	0.48	0.12	0.00153
SPSB1	103.4	0.48	0.13	0.008353
GNA11	47.0	0.48	0.13	0.004472
RASGRP1	2871.3	0.47	0.15	0.021255
ZWINT	149.4	0.47	0.14	0.017006
LOC102157484	35.4	0.47	0.13	0.008445
CENPW	53.3	0.45	0.16	0.045603
STRBP	1109.2	0.45	0.14	0.017522
PRKACB	2669.2	0.45	0.12	0.006027
SCRN3	118.0	0.44	0.14	0.019213
NFATC2	1463.9	0.44	0.11	0.001213
DYNLT3	650.8	0.44	0.11	0.003712
PWWP2A	630.0	0.44	0.12	0.005904
TSPYL4	94.8	0.43	0.11	0.002402

PTGDR	258.4	0.43	0.12	0.004805
HMGCS1	763.5	0.43	0.13	0.020729
FAM69A	240.2	0.43	0.12	0.008672
MANEAL	32.4	0.42	0.13	0.014145
AMPD3	251.8	0.41	0.12	0.013799
SCAPER	121.1	0.41	0.13	0.031756
ZNF624	198.1	0.40	0.12	0.016534
DUSP6	271.2	0.40	0.14	0.046724
CASC4	276.0	0.39	0.14	0.042842
PRKX	2765.6	0.39	0.13	0.039913
LOC102158035	2616.1	0.39	0.13	0.036001
LOC100525350	75.7	0.38	0.12	0.019964
CD3G	2563.0	0.38	0.10	0.004043
ZNF770	133.9	0.38	0.13	0.046855
CYP51	442.6	0.38	0.11	0.008693
LOC100519283	292.5	0.38	0.11	0.008771
NAB1	411.7	0.38	0.13	0.039643
RCAN3	824.3	0.38	0.11	0.007647
SLC41A1	355.5	0.37	0.10	0.003652
CCDC34	59.9	0.37	0.12	0.027517
RFC3	157.6	0.37	0.12	0.031502
CD3D	1162.5	0.37	0.11	0.014102
SLC4A8	223.7	0.37	0.13	0.042675
ACAD11	105.4	0.37	0.12	0.026193
POT1	379.7	0.37	0.13	0.049801
LMAN1	574.0	0.37	0.11	0.015539
CSNK1G3	469.5	0.37	0.11	0.0153
RTTN	246.4	0.37	0.11	0.012157
GNG2	1918.3	0.36	0.10	0.004676
UGDH	393.3	0.36	0.10	0.004923
PPIP5K1	180.7	0.36	0.10	0.0101
PCNA	790.4	0.36	0.10	0.008255
HTATSF1	351.6	0.36	0.08	0.000658
GIMAP8	3024.9	0.36	0.08	0.000391
RAD18	80.6	0.36	0.11	0.019964
ZRANB3	93.9	0.35	0.11	0.030451
FANCI	226.9	0.35	0.11	0.028101
MSMO1	297.8	0.35	0.09	0.003006
ETS1	15772.7	0.35	0.11	0.032016
LOC100737582	167.1	0.35	0.10	0.013398
NSL1	177.8	0.35	0.11	0.017892
DYRK2	1738.6	0.34	0.10	0.012519
FBXO45	169.8	0.34	0.09	0.007757
PIGF	57.3	0.34	0.11	0.033307
L3MBTL3	448.3	0.33	0.10	0.021802
GCH1	127.4	0.33	0.10	0.023723
LOC100620198	1634.4	0.33	0.10	0.025957
TMEM209	332.0	0.33	0.11	0.043227
LOC110255265	214.9	0.32	0.10	0.016956

MPHOSPH6	143.4	0.32	0.10	0.019967
SLC25A53	333.9	0.32	0.07	0.000378
SCAMP5	166.6	0.32	0.11	0.037998
PRKCQ	1621.5	0.31	0.08	0.005142
ATP6V1E2	59.4	0.31	0.11	0.043644
MDH1	661.0	0.31	0.09	0.009407
ORC6	178.0	0.31	0.11	0.049841
TRIM24	522.3	0.31	0.09	0.007966
CD40LG	97.4	0.31	0.10	0.030373
SCP2	361.9	0.31	0.10	0.026739
BCKDHB	85.7	0.31	0.10	0.035421
NSUN3	109.0	0.31	0.11	0.046193
LOC100156375	333.0	0.31	0.10	0.03707
LPIN1	519.6	0.30	0.10	0.029797
SLAIN1	243.4	0.30	0.10	0.025584
ACTR6	244.1	0.30	0.09	0.020112
PJA1	66.0	0.30	0.10	0.045436
PNRC2	2884.2	0.30	0.10	0.033129
NUAK2	627.1	0.30	0.09	0.011506
LOC110261291	153.0	0.30	0.10	0.03312
GTPBP10	203.3	0.29	0.09	0.021255
HOMEZ	154.8	0.29	0.08	0.003805
ZNF239	365.6	0.29	0.10	0.034795
TBC1D2B	1123.1	0.29	0.10	0.031707
GLMN	152.4	0.29	0.10	0.044446
YEATS4	172.3	0.29	0.10	0.033988
ITPRIPL2	913.8	0.29	0.10	0.039913
SMYD4	159.3	0.29	0.08	0.006543
ZBTB14	164.3	0.29	0.09	0.026447
GTF3C3	392.9	0.29	0.10	0.049693
STMN1	2257.5	0.28	0.09	0.023258
SLC9A6	352.9	0.28	0.08	0.007514
ITPRIPL1	234.3	0.28	0.09	0.027958
TBC1D31	137.0	0.28	0.09	0.034059
TRIM62	75.7	0.28	0.09	0.033169
USPL1	383.1	0.28	0.09	0.025814
UBR7	491.2	0.28	0.08	0.014944
MAIP1	87.2	0.28	0.09	0.01887
HGSNAT	712.0	0.27	0.07	0.005711
ZCCHC3	255.7	0.27	0.07	0.006583
STK39	747.6	0.27	0.09	0.03628
CD2	3011.8	0.27	0.09	0.041954
MRPL47	189.0	0.27	0.09	0.041017
CEPT1	648.0	0.26	0.09	0.03605
TMEM126B	155.0	0.26	0.09	0.035355
GEMIN2	251.1	0.26	0.08	0.013246
RFC5	388.8	0.26	0.08	0.021404
RBBP4	3323.2	0.26	0.07	0.00605
ZMIZ1	1962.0	0.26	0.09	0.041286

FYN	2933.0	0.26	0.08	0.029113
TM9SF3	1450.3	0.26	0.09	0.043266
SMIM10L1	617.8	0.26	0.08	0.015472
PCYOX1	525.5	0.26	0.09	0.04341
ABT1	372.0	0.26	0.06	0.000841
NOP58	934.2	0.26	0.07	0.003966
TIMM21	129.4	0.26	0.08	0.02582
APOBEC3B	472.1	0.26	0.09	0.038669
ASF1A	159.4	0.25	0.08	0.032393
PRIM2	235.4	0.25	0.08	0.018257
DERA	341.5	0.25	0.08	0.01964
EIF3J	746.6	0.25	0.07	0.011536
ELK3	519.9	0.25	0.09	0.046106
GATC	217.2	0.25	0.07	0.011569
TXNDC11	558.7	0.25	0.07	0.007013
ERCC8	191.3	0.25	0.08	0.033641
BAZ1B	1989.6	0.25	0.09	0.046379
HNRNPR	2149.1	0.25	0.09	0.049463
FAM104A	800.2	0.24	0.08	0.041954
GLUD1	2432.4	0.24	0.06	0.005059
ARMT1	201.8	0.24	0.08	0.035043
LSM8	248.5	0.24	0.07	0.017876
PAK1IP1	204.2	0.24	0.08	0.035642
KAT7	1190.9	0.24	0.07	0.014197
MBIP	363.8	0.24	0.07	0.010527
PHAX	378.5	0.24	0.08	0.044112
CDC5L	1205.3	0.24	0.08	0.026507
HMGB1	5129.6	0.24	0.08	0.025676
MPHOSPH10	354.8	0.23	0.08	0.035421
TMED7	357.9	0.23	0.07	0.010469
NSRP1	369.0	0.23	0.08	0.039289
ZNF706	659.1	0.23	0.07	0.017725
VAMP4	412.6	0.23	0.07	0.020829
CASP6	293.0	0.23	0.08	0.041871
SFT2D3	111.7	0.23	0.08	0.048255
FAM210A	303.8	0.23	0.08	0.047242
TAF4	447.5	0.23	0.07	0.020665
TMEM230	857.1	0.23	0.08	0.034688
ESYT1	4102.2	0.23	0.08	0.041014
WASHC3	330.5	0.23	0.08	0.033081
FBXO25	403.3	0.23	0.07	0.030962
TMEM106C	644.7	0.23	0.08	0.042049
PIP4K2C	588.2	0.23	0.07	0.025417
ICMT	585.2	0.22	0.07	0.014218
LRR1	212.4	0.22	0.07	0.037877
LOC100524613	1053.8	0.22	0.06	0.005439
PRDM4	261.3	0.22	0.07	0.028062
TIGD2	233.4	0.22	0.07	0.030373
INTS10	463.6	0.22	0.07	0.039226

SNX12	481.6	0.22	0.07	0.017619
C2H11orf58	760.4	0.22	0.07	0.037626
PRPF8	5949.5	0.22	0.06	0.008503
PDP2	310.7	0.22	0.07	0.026623
TCEA1	1177.1	0.22	0.07	0.029939
CCDC93	705.0	0.21	0.07	0.034847
SRSF3	3087.5	0.21	0.07	0.021234
PPP1R8	582.9	0.21	0.07	0.049693
HS2ST1	263.4	0.20	0.07	0.026357
RNMT	739.2	0.20	0.06	0.028149
TSN	737.6	0.20	0.06	0.017598
R3HDM1	1231.9	0.20	0.06	0.031707
GGCX	411.1	0.20	0.07	0.040317
DNAJC2	477.5	0.19	0.07	0.036001
PREP	583.3	0.19	0.06	0.027959
ERCC3	647.3	0.19	0.07	0.040183
HNRNPD	3198.3	0.19	0.06	0.043278
INIP	499.5	0.18	0.06	0.036977
UNG	550.9	0.18	0.06	0.041625
FIP1L1	806.4	0.18	0.06	0.045436
ANKLE2	591.5	0.18	0.06	0.01955
NUP88	927.0	0.17	0.06	0.027959
IKZF1	4524.8	0.16	0.05	0.026625
EIF4ENIF1	650.7	0.16	0.05	0.045154
VPS53	666.1	0.16	0.05	0.039451
SEC31A	2168.1	0.15	0.05	0.039451
USP30	474.9	0.15	0.05	0.04759
HMG20A	1132.9	0.14	0.05	0.024151

Supplementary table 3 Amplicon sequence variants that were significantly more abundant in jejunal content collected from pigs 12 d post-weaning vs samples at weaning

ASV	T1 Mean	T2 Mean	log2 FC	log2 FC SE	Adjusted P	Phylum	Class	Order	Family	Genus	Species
ASV5	3139.0	27444.0	4.28	0.97	8.17E-05	Firmicutes	Bacilli	Lactobacillales	Streptococcaceae	Streptococcus	porcorum
ASV9	10.64	11639.8	6.42	2.28	0.013931	Firmicutes	Bacilli	Lactobacillales	Lactobacillaceae	Pediococcus	NA
ASV13	4.26	31351.1	6.91	1.56	8.17E-05	Firmicutes	Bacilli	Erysipelotrichales	Erysipelatoclostridiaceae	Catenibacterium	mitsuokai
ASV16	286.93	2032.30	2.82	1.27	0.04961	Actinobacteriota	Actinobacteria	Micrococcales	Micrococcaceae	Rothia	nasimurium
ASV19	144.16	16804.4	4.33	1.36	0.004897	Actinobacteriota	Coriobacteriia	Coriobacteriales	Coriobacteriaceae	Collinsella	aerofaciens
ASV26	9.01	4120.10	8.74	1.51	2.82E-07	Firmicutes	Clostridia	Lachnospirales	Lachnospiraceae	Blautia	NA
ASV58	2.39	1065.98	6.48	2.38	0.018252	Firmicutes	Clostridia	Peptostreptococcales-Tissierellales	Peptostreptococcales-Tissierellales_fa	Anaerococcus	NA
ASV65	30.85	484.40	5.05	1.37	0.000983	Firmicutes	Bacilli	Staphylococcales	Gemellaceae	Gemella	NA
ASV66	17.03	574.71	5.29	1.64	0.004343	Firmicutes	Clostridia	Oscillospirales	Ruminococcaceae	Subdoligranulum	NA
ASV73	0.97	487.30	9.03	1.66	1.40E-06	Firmicutes	Bacilli	Erysipelotrichales	Erysipelotrichaceae	Holdemanella	NA
ASV79	24.86	182.99	4.84	1.94	0.027604	Actinobacteriota	Actinobacteria	Actinomycetales	Actinomycetaceae	Trueperella	NA
ASV80	9.03	220.58	6.33	1.54	0.000241	Firmicutes	Clostridia	Peptostreptococcales-Tissierellales	Peptostreptococcaceae	Peptostreptococcus	NA
ASV83	7.57	464.96	8.06	2.04	0.000391	Firmicutes	Bacilli	Staphylococcales	Staphylococcaceae	Staphylococcus	NA
ASV93	2.84	375.72	7.08	1.50	4.43E-05	Firmicutes	Clostridia	Peptostreptococcales-Tissierellales	Anaerovoracaceae	Family_XIII_AD3011_group	NA
ASV97	2.25	136.73	8.78	2.93	0.008229	Bacteroidota	Bacteroidia	Bacteroidales	Rikenellaceae	Rikenellaceae_RC9_gut_group	NA
ASV99	5.87	262.40	4.91	1.91	0.023228	Firmicutes	Clostridia	Clostridiales	Clostridiaceae	Clostridium_sensu_stricto_1	NA
ASV102	0.24	282.63	9.08	2.17	0.000188	Firmicutes	Bacilli	Lactobacillales	Leuconostocaceae	Weissella	NA
ASV105	5.79	433.38	5.55	1.37	0.000274	Euryarchaeota	Methanobacteria	Methanobacteriales	Methanobacteriaceae	Methanobrevibacter	NA
ASV112	0.27	332.95	8.99	2.15	0.000188	Firmicutes	Clostridia	Oscillospirales	Butyricicoccaceae	UCG-008	NA
ASV125	9.34	3993.85	5.61	2.42	0.039507	Firmicutes	Clostridia	Christensenellales	Christensenellaceae	Christensenellaceae_R-7_group	NA
ASV132	1.45	158.93	7.12	2.72	0.022543	Firmicutes	Bacilli	Lactobacillales	Carnobacteriaceae	Granulicatella	NA
ASV136	0.00	209.90	26.39	2.09	1.43E-34	Firmicutes	Clostridia	Lachnospirales	Lachnospiraceae	Marvinbryantia	NA
ASV138	18.83	67.20	4.56	1.75	0.022543	Actinobacteriota	Coriobacteriia	Coriobacteriales	Atopobiaceae	Olsenella	umbonata
ASV140	1.89	105.41	5.82	1.92	0.007718	Firmicutes	Clostridia	Lachnospirales	Lachnospiraceae	Coprococcus	comes
ASV141	1.97	176.67	8.26	1.80	5.79E-05	Firmicutes	Bacilli	Erysipelotrichales	Erysipelotrichaceae	Solobacterium	NA
ASV158	2.85	75.66	5.95	1.69	0.001592	Firmicutes	Clostridia	Peptostreptococcales-Tissierellales	Anaerovoracaceae	Mogibacterium	NA

ASV160	0.86	177.71	7.70	2.00	0.000537	Firmicutes	Clostridia	Lachnospirales	Lachnospiraceae	Dorea	formicigenerans
ASV170	0.00	97.22	7.71	2.93	0.022543	Firmicutes	Clostridia	Peptostreptococcales-Tissierellales	Peptostreptococcales-Tissierellales_fa	Finegoldia	NA
ASV173	2.27	56.03	4.59	1.92	0.034009	Actinobacteriota	Coriobacteriia	Coriobacteriales	Atopobiaceae	Atopobium	fossor
ASV180	0.50	163.38	8.32	1.79	5.21E-05	Firmicutes	Bacilli	Erysipelotrichales	Erysipelotrichaceae	Catenisphaera	NA
ASV185	0.00	90.05	8.06	2.06	0.000456	Firmicutes	Clostridia	Lachnospirales	Lachnospiraceae	Lachnospiraceae_NC2004_group	NA
ASV197	3.33	30.22	6.60	2.55	0.022543	Actinobacteriota	Actinobacteria	Actinomycetales	Actinomycetaceae	Arcanobacterium	NA
ASV214	2.02	33.45	5.57	1.50	0.000931	Firmicutes	Clostridia	Peptostreptococcales-Tissierellales	Peptostreptococcales-Tissierellales_fa	Parvimonas	NA
ASV222	0.43	26.40	5.96	1.91	0.00577	Firmicutes	Clostridia	Lachnospirales	Lachnospiraceae	Anaerostipes	NA
ASV223	0.00	51.46	8.81	2.43	0.00111	Firmicutes	Clostridia	Peptococcales	Peptococcaceae	Peptococcus	NA
ASV241	0.00	440.11	8.90	1.96	5.97E-05	Firmicutes	Clostridia	Oscillospirales	Ruminococcaceae	Ruminococcus	NA
ASV251	0.00	32.81	7.14	2.76	0.022543	Firmicutes	Clostridia	Peptostreptococcales-Tissierellales	Peptostreptococcales-Tissierellales_fa	Peptoniphilus	NA
ASV264	0.00	66.09	9.15	2.16	0.000176	Firmicutes	Clostridia	Oscillospirales	Ruminococcaceae	Incertae_Sedis	NA
ASV269	0.00	36.08	7.17	2.94	0.030694	Firmicutes	Clostridia	Peptostreptococcales-Tissierellales	Peptostreptococcaceae	Intestinibacter	NA
ASV288	1.80	419.70	6.39	2.51	0.024701	Firmicutes	Clostridia	Oscillospirales	Oscillospiraceae	UCG-005	NA
ASV383	1.03	29.87	6.61	2.79	0.035449	Firmicutes	Clostridia	Peptostreptococcales-Tissierellales	Peptostreptococcales-Tissierellales_fa	Helcococcus	NA
ASV583	0.00	5.99	5.77	2.50	0.039507	Patescibacteria	Saccharimonadia	Saccharimonadales	Saccharimonadaceae	Candidatus_Saccharimonas	NA

Supplementary table 4 Initial seeds of normalized expression of genes selected for activation of MHC class I and IFNγ in jejunal Payer's patches collected from pigs 12 d post-weaning, in the different cluster groups.

	Cluster	
Gene	1	2
AZGP1	2.010	0.069
B2M	1.967	-1.615
BATF2	-0.495	-0.561
CD1D	-0.956	-0.962
CD1E	-1.052	1.863
CD3D	-1.319	-0.606
CD3E	-0.879	-0.281
CD3G	-1.155	-1.025
CD8A	-0.669	-0.624
CHMP1B	1.664	-1.683
CHMP2A	0.058	-0.612
CHMP2B	2.591	-1.274
CHMP3	0.925	-1.234
CHMP5	1.225	-1.545
ERAP1	1.020	-2.166
ERAP2	0.813	-2.653
FCGRT	0.848	-1.317
HFE	1.474	-1.463
IRF1	-0.620	0.032
LAPTM4B	1.779	-1.467
LEPROT	1.226	-1.543
LEPROTL1	0.956	-1.222
LYST	0.284	1.084
MVB12A	0.188	-0.627
PSMB10	-0.624	-1.142
PSMB9	-0.159	-1.590
RAB27A	-1.314	-0.194
RAB27B	1.112	-1.346
RILP	0.617	-0.658
RUBCN	1.921	-0.489
SLA_2	1.596	-1.500
SORT1	2.589	-1.383
TAP1	1.313	-1.173
TAP2	0.816	-1.573
TAPBP	0.220	-0.646
TMEM50A	1.451	-1.477
TMEM50B	1.866	-1.572
UBD	-0.446	-0.373
UVRAG	-1.132	0.621
VPS36	1.338	-1.722
VPS37A	3.083	-1.274
VPS37C	2.338	-0.860

VPS4B	1.781	-2.184
XCL1	0.269	-1.807

Supplementary table 5 Ingredients and calculated composition of the pre-starter feed expressed on as fed

Item	Units	Content
Ingredients		
Bakery former food	%	20.00
Barley	%	15.00
Soybean Protein Concentrate	%	13.50
Wheat, soft	%	12.20
Maize	%	11.75
Whey, sweet, dehydrated, skimmed	%	9.00
Wheat middlings	%	5.00
Spray dried porcine plasma	%	3.00
Lard	%	2.00
Beet pulp, dehydrated	%	1.50
Dicalcium phosphate anhydrous	%	1.40
Dextrose	%	1.20
Medium chain free fatty acid mixture	%	1.00
Organic acid mixture	%	1.00
L-Lysine HCl	%	0.55
Calcium carbonate	%	0.53
Sodium chloride	%	0.30
Vitamin and trace mineral mixture[1]	%	0.30
DL-Methionine	%	0.28
L-Threonine	%	0.28
L-Valine	%	0.11
L-Tryptophan	%	0.10
Calculated values[2]		
Metabolizable energy	Kcal/kg	3340
Crude Protein	%	18.00

Crude Fat	%	6.44
Crude Fibre	%	2.75
Ash	%	5.71
Lysine	%	1.34
Cysteine	%	0.30
Methionine	%	0.51
Threonine	%	0.95
Tryptophan	%	0.29
Valine	%	0.99

[1] Provided per kg of diet: vitamin A (retinyl acetate): 7,500 IU; vitamin D3 (cholecalciferol): 1,000 IU; vitamin E (DL-α_tocopheryl acetate): 80 mg; vitamin K (menadione sodium bisulfite): 1 mg; riboflavin, 2.3 mg; calcium-D-pantothenate: 9.0 mg; niacin: 17.5 mg; pyridoxine hydrochloride: 1 mg; folic acid: 0.5 mg; biotin: 0.10 mg; thiamine: 1.45 mg; vitamin B12: 15 µg; ferrous sulphate monohydrate: 302.8 mg; zinc oxide: 118.2 mg; manganous sulphate monohydrate: 18.48; copper sulphate: 347.6; sodium selenite: 0.55 mg; potassium iodide: 1.31 mg.

Supplementary table 6 List of genes of which it was used the expression, for the clustering of samples collected in the post-weaning period

AZGP1	B2M	BATF2	CD1D	CD1E	CD3D	CD3E	CD3G	CD8A
CHMP1B	CHMP2A	CHMP2B	CHMP3	CHMP5	ERAP1	ERAP2	FCGRT	HFE
IRF1	LAPTM4B	LEPROT	LEPROTL1	LYST	MVB12A	PSMB10	PSMB9	RAB27A
RAB27B	RILP	RUBCN	SLA_2	SORT1	TAP1	TAP2	TAPBP	TMEM50A
TMEM50B	UBD	UVRAG	VPS36	VPS37A	VPS37C	VPS4B	XCL1	

Supplementary Fig.1

Effect of the attribution of each pig to the specific cluster group for MHC class I and IFNγ activation in JPPs, on Alpha diversity of-his jejunal microbiota

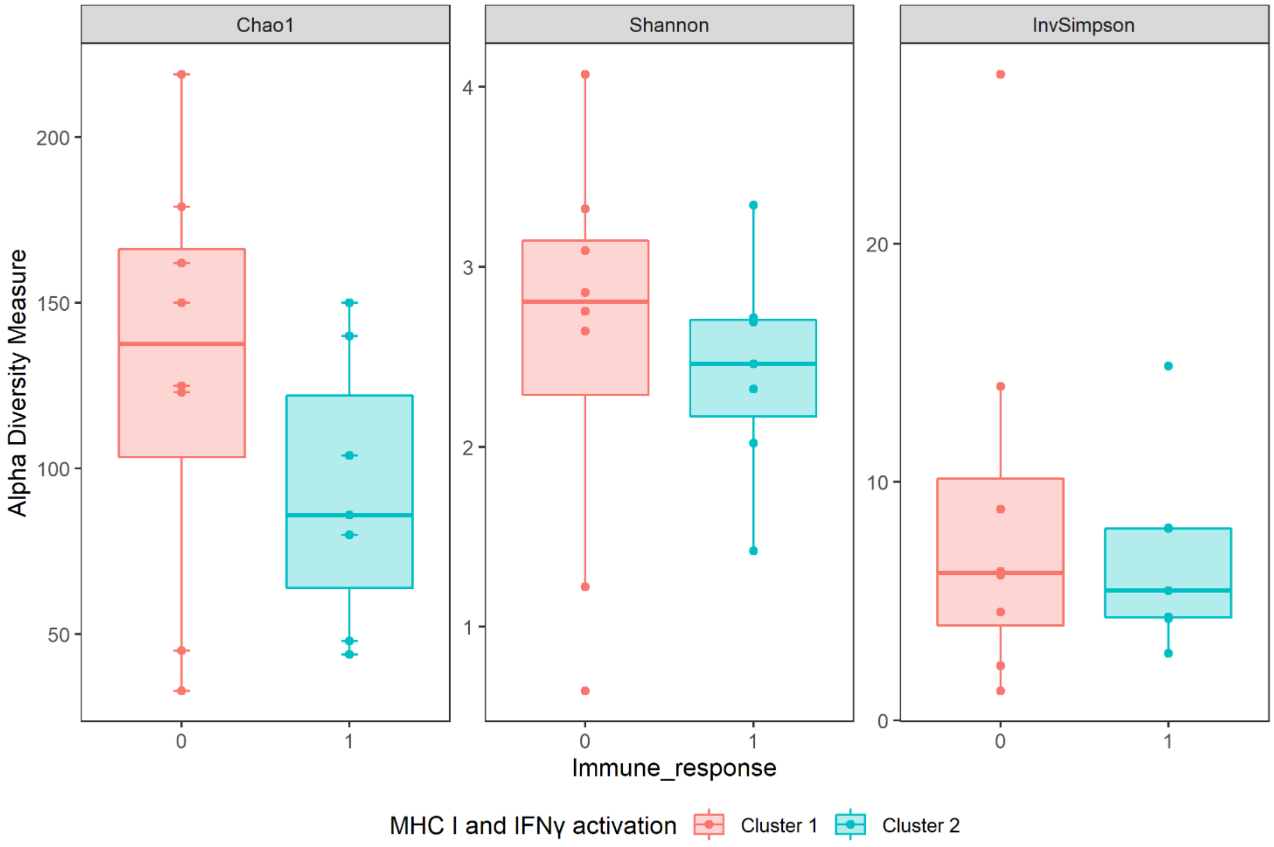

Supplementary Fig.2

Effect of the attribution of each pig to the specific cluster group for MHC class I and IFNγ activation in JPPs, on Beta-diversity of his jejunal microbiota

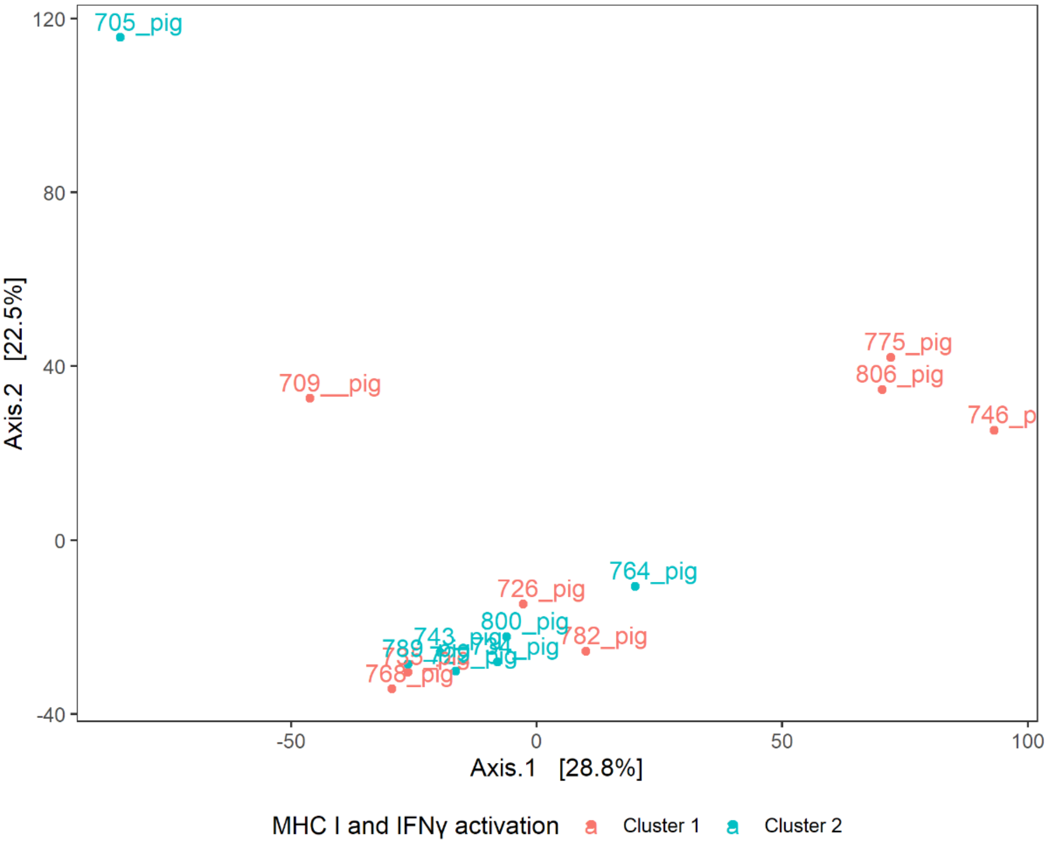

Supplementary Fig.3

Plot of Principal Component Analysis of the normalized expression of genes selected for activation of MHC class I and IFNγ in jejunal Payer's patches collected from pigs 12 d post-weaning.

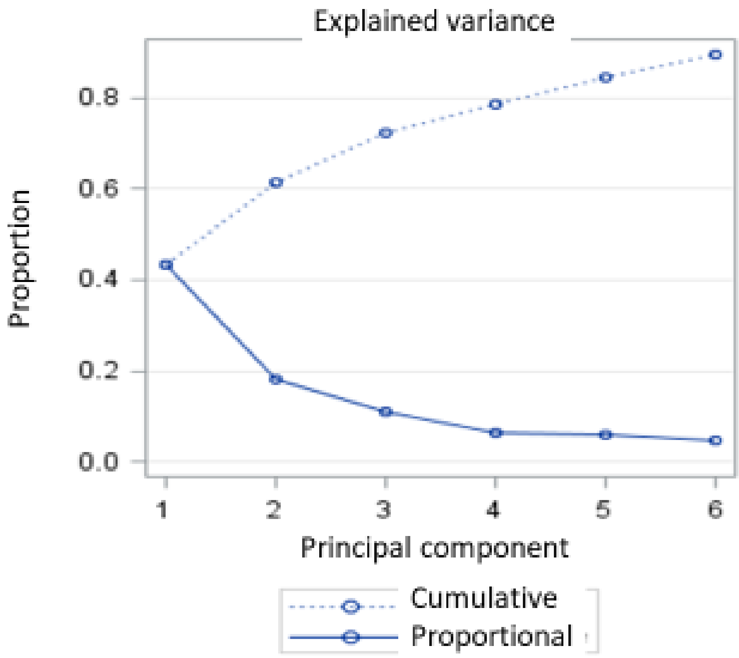

Supplement: Supplementary file 1 — Supplementary Information. [file 41598_2022_5707_MOESM1_ESM.pdf]
